# Acceptability, feasibility and fidelity of the culturally adapted version of Unplugged (“Yo Se Lo Que Quiero”), a substance use preventive program among adolescents in Chile: a pilot randomized controlled study

**DOI:** 10.1186/s12889-024-19499-2

**Published:** 2024-07-29

**Authors:** Gabriel Salgado, Jorge Gaete, Sofía Gana, Daniela Valenzuela, Ricardo Araya

**Affiliations:** 1 Magíster en Epidemiología, Facultad de Medicina, Universidad de los Andes, Santiago, Chile; 2 Millennium Nucleus to Improve the Mental Health of Adolescents and Youths (Imhay), Santiago, Chile; 3 Centro de Investigación en Salud Mental Estudiantil (ISME), Escuela de Educación, Facultad de Ciencias Sociales, Universidad de los Andes, Santiago, Chile; 4 Doctorado en Ciencias del Desarrollo y Psicopatología, Laboratorio de Ciencias Cognitivas, Facultad de Psicología, Universidad del Desarrollo, Santiago, Chile; 5 Department of Health Service & Population Research, David Goldberg Centre, King´s College London, London, UK

**Keywords:** Adolescents, Substance abuse, Prevention, Acceptability, Feasibility, Fidelity

## Abstract

**Introduction:**

The consumption of alcohol, tobacco, and cannabis is a public health problem that impacts the cognitive, social, and emotional development of adolescents. Prevention strategies such as the “Unplugged” program are effective in delaying the progression of daily smoking and episodes of drunkenness among adolescents. “Yo Se Lo Que Quiero” (YSLQQ) corresponds to the adaptation of this program to the Chilean context. This study assesses the acceptability and feasibility of implementing this program to the local reality.

**Material and methods:**

This was a cluster-randomized controlled pilot study conducted on six public schools. All consented students attending 6th, 7th, and 8th grades (*n* = 1,180) participated in the study. The schools were randomly assigned to one of two conditions in a 1:1 ratio: (1) the “YSLQQ” intervention group (*n* = 526), and (2) the Control group (*n* = 654). The program consisted of a 12-hour class-based curriculum based on a comprehensive social-influence approach delivered by a trained facilitator. The acceptability and feasibility were assessed in the intervention group at the end of the intervention using questionnaires answered by students and facilitators. The quality and fidelity of the program were evaluated during the implementation using self-ported surveys answered by the facilitators and the assessment of video-recorded sessions rated by external observers. Finally, a pre-test and a post-test survey assessing past and current substance use and risk and protective factors were conducted before and immediately after the program’s implementation.

**Results:**

A high proportion of students (49.6%) liked the sessions. 79.2% reported that the YSLQQ helped them learn about the dangers of substances, while 65.8% reported having more skills to avoid substance use in the future. Regarding students’ satisfaction with YSLQQ, 62.9% reported being happy or very happy with the program. Facilitators reported implementing the intervention according to the manual in 73.9% of sessions. Regarding substance use, students who participated in the intervention groups reported a significant reduction in drunkenness in the last year and last 30-day prevalence and also a significant reduction in a lifetime and 30-day prevalence of cannabis use when compared with those students in the control group.

**Conclusions:**

Our results suggest that YSLQQ has adequate acceptability and feasibility to be implemented in the Chilean context, and there were promising results in reducing drunkenness and cannabis use. Future research should confirm these results in a larger RCT study.

**Trial registration:**

The trial was registered in ClinicalTrials.gov, NCT04566627; registration date: 01/03/2019

**Supplementary Information:**

The online version contains supplementary material available at 10.1186/s12889-024-19499-2.

## Introduction

Substance use and drug-related disorders are important public health problems. Mental and substance use disorders worldwide accounted for over 225 million DALYs of disease burden in 2019 [1]. Specifically, alcohol (4.2% DALYs) and substance use account for (1.3% DALYS) 5.5% of the total burden of disease [2]. In Chile, the burden of disease attributable to substance use is 461.0 DALYs, above the global average of 421 DALYs. Regarding alcohol consumption, the burden of disease in Chile is 1,219 DALYs, just below the average worldwide of 1,352 DALYs [2].

Substance use is a significant health issue with well-documented consequences. Tobacco smoking is linked to respiratory problems, cardiovascular diseases, cancer, cognitive issues, and mental health problems such as depression and anxiety [3–5]. Alcohol use among adolescents is associated with memory, attention, and other cognitive problems [6–8]. Cannabis smoking can cause chronic bronchitis, damage airway cells, and negatively affect brain development, leading to reduced intellectual capacity, attention, memory, and abstract thinking issues, as well as increased risks for psychotic symptoms, anxiety, and depression [9–14]. Early cannabis use amplifies these risks [11].

In Chile, substance use among adolescents is a major concern. One in four students from 8th to 12th grade has smoked cigarettes in the last month, 31.1% have consumed alcohol, and one in three has used cannabis [15]. Chile has the highest rates of tobacco and cannabis use among secondary students in the Americas [16].

Over the past 40 years, Chile has implemented various preventive programs to combat substance use among school-aged children. Initially, the Consejo Nacional para el Control de Estupefacientes (CONACE) spearheaded these initiatives, later replaced by the Servicio Nacional para la Prevención y Rehabilitación del Consumo de Drogas y Alcohol (SENDA) in 2011. Key programs include the “Programa Previene” initiated in 1997 and the “Continuo Preventivo” introduced in 2009, which offered age-specific activities: “En busca del Tesoro” for preschoolers, “Marori y Tutibú” for 1st to 4th graders, “Quiero Ser” for 5th to 8th graders, and “Yo Decido” for 9th to 12th graders [17]. Despite these efforts, none of the programs have been rigorously evaluated through randomized controlled trials, and substance use among Chilean adolescents remains high.

## Unplugged, a substance use preventive program: aims, structure, and effectiveness worldwide and in Latin America

Several systematic reviews have found that universal school-based interventions, especially those that include anti-drug information, refusal skills, self-management skills, and social competence training, effectively reduce legal and illegal substance use among adolescents [18–20]. For example, a recent systematic review found that the “Unplugged” program appears to have the best evidence of effectiveness [21].

The “Unplugged” preventive program was designed based on the Comprehensive Social Influence Model [22, 23], incorporating the educational approach to life skills and normative beliefs about substances. This program aims to delay the onset of substance use and the transition from occasional to habitual use. It is focused on tobacco, alcohol, and cannabis consumption. Trained teachers deliver it in 12 sessions or units over one academic year, and the program includes teaching factual knowledge and attitudes regarding substance use (4 sessions) and training in interpersonal skills (4 sessions) and intrapersonal skills (4 sessions). It has been evaluated in several European countries with positive short- and long-term effects [24, 25] and in other countries, such as Nigeria and Brazil, with mixed results. In Nigeria, the evaluation study was conducted on students attending first grades of secondary schools, and the intervention reduced the prevalence of recent alcohol use and the progression to more intense alcohol use [26]. However, there were no significant positive effects of tobacco and cannabis use. In Brazil, three versions of Unplugged were tested. The first version (2013) was studied in a non-randomized trial. The results suggested that the program may have played a role in a decrease in recent cannabis use and binge drinking practices [27]. In the second version, in 2014 and 2015, after a cultural adaptation and important content changes were made in the Unplugged classes, the program was renamed #Tamojunto [28]. In a large randomized controlled trial (RCT) to evaluate the effectiveness of the #Tamojunto version, an iatrogenic effect on the initiation of alcohol use was found after 9 and 21 months of follow-up [29]. These unexpected results were attributed to cultural adaptations of the alcohol-related components of the program. The #Tamojunto version excluded the original Unplugged components that reinforced non-alcohol use and replaced them with a harm reduction approach [30]. Based on the negative results found in the #Tamojunto RCT, a new adaptation of the program, now named #Tamojunto2.0, reinstating the original components of the Unplugged program and removing the components related to harm reduction. The results of this new adaptation found that #Tamojunto 2.0 reduced alcohol initiation; however, no effects were found on the prevention of any other substance initiation or prevalence of past-month substance use among 8th-grade students in Brazil [31].

## The adaptation process of Unplugged in Chile

This program was adapted following the guidelines in the literature on replicating evidence-based prevention programs in new contexts and the discussion on the deep and surface structure of interventions [32, 33]. The deep structure of intervention includes the intervention theory of change. According to the leading theories of the social influence approach (social learning and social norms), substance use initiation results from social influence, from which adolescents can derive erroneous perceptions of frequency and acceptability of use. Normative education and resistance skills training included in Unplugged curricula are thought to reduce the effect of social influence by modifying attitudes, beliefs, and normative perceptions, finally supporting the development of general social skills and skills to resist social pressures. The intervention surface structure consists of the parts that improve the face validity of the intervention with participants, language, material, activities, and communication channels. Researchers stress maintaining the deep structure while allowing surface structure modifications in new contexts. The degree of this adaptive change can vary from minimal to complete adaptation [33]. We decided to define a minimally adapted edition of the Unplugged program with only surface structure changes. The deep structure of Unplugged remained unchanged in the Chilean version. The surface structure of the Unplugged materials was screened by two psychologists from our research group and one teacher. This screening showed that the intervention materials and activities were acceptable and culturally relevant, with only minimal changes necessary. The original European developers were informed about all the modifications proposed for the Chilean version, which was approved. Specific modifications to the materials involved changes in the language, pictures included in the materials, and some stories used during the student lessons.

No previous evaluation of this program has been carried out in Chile. Despite the severe substance use problem among adolescents, no studies have tested the effectiveness of any universal school-based substance use preventive program in Chile.

This study aimed to develop a culturally appropriate version of Unplugged, henceforth “Yo Sé Lo Que Quiero” (YSLQQ), and to assess the acceptability and feasibility of its implementation among adolescents in low-income primary schools in Santiago, Chile. We also explored the program’s efficacy in preventing tobacco, alcohol, and cannabis use.

## Methods

### Study design and participants

This was a cluster randomized controlled pilot study. Our target sample was vulnerable Santiago schools, with students from low—to low-middle-income families. Most students in Chile attend vulnerable schools (91%) [34]. These students usually receive a low-quality education, and their academic performance on standardized tests is the lowest compared with students coming from more affluent families [35]. Additionally, substance use also has this unequal distribution, and the impact is greatest on the lives of the most needed adolescent population [36]. These reasons and the possibility of providing evidence of research-based interventions for policymakers to help most of the adolescent population have motivated the research team to concentrate the effort on the most vulnerable population. Our sample frame was the registry of schools with primary education (Year 1 to Year 8) located in Santiago, with a vulnerability index (School Vulnerability Index – National System of Equality Allocation (IVE-SINAE)) ≥ 70%. The IVE-SINAE is built considering several students’ and parental variables: health, family income, and receiving state benefits. This percentage means the proportion of students in a school who are most needed.

The study population consisted of students attending Year 6 (around 12 years old), 7, and 8 (about 14 years old). We target adolescents aged 12–14 because this is when adolescents start experimenting with substances (especially smoking and alcohol) and because this was the age range of students included in the original evaluation of Unplugged in Europe [37, 38].

#### Inclusion criteria for schools

1. To have at least two classes in the grade under study. This criterion is based on the reality that most of the schools in Chile have between 2 and 3 classes per grade level, and the results of this intervention may facilitate the generalization to other schools.
2. To be a mixed-sex school. Most of the schools in Chile are in this condition.
3. To accept participation under the conditions of the study, mainly to accept being part of the control group after randomization. Our research team has found that schools, students, and their parents or caregivers are less interested in participating in a study when there is a chance of being allocated to a control group. However, information on feasibility (e.g., response rate to the invitation, response rate of informed consent) is very valuable for planning an RCT at a large scale.

#### Exclusion criterion

Schools implementing other interventions with strong and manualized packages targeted at the same grades. Very few schools in Chile have implemented manualized preventive programs. However, we have introduced this criterion for two reasons: (1) Known that it is difficult to implement preventive programs in schools, but if the school already has invested in this kind of program, we consider that they should concentrate their efforts in keeping with that initiative; (2) The comparison group will be the situation found in most of the schools in Chile, that is, the presence of isolated preventive actions.

#### Ethical issues

Data were collected following the Declaration of Helsinki. This study was approved by the ethics committee CEC201734 (08/07/2018).

### Sample size

Since this is a pilot study, it is not appropriate to calculate a sample size for establishing the effectiveness of the intervention [39]. However, we have calculated a suitable number of adolescents for this study. According to recommendations for feasibility studies, a minimum of 30 participants per arm is useful to estimate the parameters for future sample size calculations [40]. In addition, we wanted to ensure some variability, so we proposed to include three schools per arm, with three grades (Year 6, 7, and 8), with at least 60 students per grade in each arm. However, to test the efficacy or the effectiveness of school-based preventive interventions, it is necessary to work with a large sample size [41], so no definitive conclusions can be drawn from exploring the efficacy presented in this study.

### Procedure

First, we reviewed the records of schools provided by the Ministry of Education (MINEDUC) that met the inclusion and exclusion criteria (*n* = 126). We contacted schools at the end of 2018 and the beginning of the academic year (March) in 2019 until we reached the aimed number (*n* = 6). Most schools did not respond to our email invitation (*n* = 112) during recruitment. Fourteen schools responded and manifested some interest in the study. However, when contacted again by telephone or personally, two refused to participate in the study, six did not give any further response, and six agreed. We ended the recruitment process when we reached the number of schools originally proposed.

The evaluations were carried out during class hours, according to a schedule pre-established by the schools; the evaluators answered questions and clarified doubts for the students before they answered the instruments. The baseline assessment was conducted between March and April 2019. The intervention was implemented between May and October 2019. A post-intervention assessment was conducted in October 2019.

### Randomization and allocation

Once the schools agreed to participate in this research, they were randomly allocated to (1) the YSLQQ group or (2) the Control Group. The randomization was carried out by an independent statistician using the platform www.random.org. After this allocation, each school was informed of the assigned group, and the research group started coordinating the sending of the written and informed consent to parents and caregivers, scheduling the baseline assessment, and, for the YSLQQ group, organizing the training for school staff.

### Blind condition

In this research, schools, facilitators, and training staff were not blind to the group allocation because of the nature of the intervention. However, evaluators and the statistician who conducted the final analyses were blinded.

### Intervention group: “Yo Se Lo Que Quiero”

#### YSLQQ curriculum

The program consists of 12 45-minute sessions taught weekly by previously trained facilitators. Briefly, the curriculum is taught using interactive techniques. It aims to develop and enhance personal and social skills (critical and creative thinking, relationship skills, assertiveness, refusal skills, verbal and non-verbal communication, managing emotions, coping skills, empathy, problem-solving, and decision-making skills) with a specific focus on normative education (e.g., the correction of normative beliefs about substances and of the incorrect perception of the prevalence of use among peers). The titles of the 12 sessions are as follows: [1] What is I Know What I Want? [2], Inside or outside the student group [3], Alcohol; risks and protection [4], Will it be as you think [5]? What to expect from tobacco? [6], With your own voice [7], Assert yourself in the face of pressure [8], On stage! [9], ) Know more, risk less [10], Strategies to face difficulties [11], Solve problems and face decisions [12], Establish objectives. For more information regarding each session, see Supp 1.

The training consisted of a three-day workshop held by Chile’s Unplugged master trainer coupled with the master trainer of the EU-Dap Faculty in February 2019. All sessions were reviewed in the training, and four were executed by the training participants. Chile´s Unplugged master trainer modeled two sessions; the rest were examined in depth. The objective of the training was to exemplify an experiential program through modeling activities.

The school curriculum was taught in the selected Chilean schools from April to November 2019. To standardize the program’s implementation, a Teacher Manual, an adequate number of quiz card sets, and a Student Booklet were provided to teachers and students. The whole adaptation, implementation, and evaluation processes were conducted for the research team without the involvement of any state agencies.

#### Recruitment of facilitators

School teachers in the Chilean system feel that the workload is very high, and they consider it challenging to implement the curriculum by themselves; therefore, the program was implemented by facilitators. These facilitators were educators with at least two years of experience working in the classrooms, hired and trained by the research team in a 3-day workshop. Additionally, ensuring the implementation follows a high fidelity and adherence to the manual was very important. To ensure the fidelity of the implementation, we provided supervision to the facilitators, which consisted of weekly face-to-face meetings during the school year (2 h each) with the research team to prepare lessons and to monitor the implementation, and a direct line (phone and e-mail) to discuss and solve specific problematic situations or doubts. Finally, in Chile, a certain number of school teachers change schools over the years, and they know which class they will be only at the end of February before the school year starts. This is one of the reasons for scheduling teachers, facilitators, and school staff training in February in the Chilean schools.

#### Supervision of facilitators

The facilitators were supervised by the team coordinator in weekly meetings in which the implementation of the content of each of the sessions, the methodological strategies used, and the problems that arose were reviewed.

### Control group

The control group received the usual teaching activities regarding substance use prevention, which were implemented in the schools as a usual practice.

### Outcomes

#### Primary outcome

##### Acceptability, feasibility, fidelity, and quality of implementation

##### Acceptability

Acceptability was assessed by establishing how this intervention program was received by the students and to what extent this intervention responds to the needs of this target population. In particular, acceptability was assessed through a questionnaire that was answered by each student who participated in the intervention. The research team developed this questionnaire, and its English and Spanish versions are found in Suppl 2. This questionnaire included five sections related to the perception and experience lived in the sessions (4 items), implementation of the program “YSLQQ” (5 items), effects and usefulness of the YSLQQ program (7 items), satisfaction with YSLQQ (4 items), and satisfaction with the duration of the sessions (3 items), each of these questions had a Likert scale (1 “Strongly disagree” to 5 “Strongly agree”). Additionally, we included open-ended questions related to the positive and negative aspects of the intervention and what modifications to the YSLQQ program they suggest.

##### Feasibility

The feasibility of the recruitment process was measured using a registry of consent and questionnaire response rates. The feasibility of the intervention implementation was measured using a registry of the number of sessions implemented.

##### Fidelity and quality of the implementation

Fidelity was assessed using a self-report questionnaire answered by each of the facilitators, in addition to a questionnaire answered by an external observer who rated the recording of the sessions performed by the facilitators. The research team developed this questionnaire, and its English and Spanish versions are found in Suppl 3. This questionnaire included questions related to the program, how much the facilitators implemented the session activities according to the manual, session times, classroom climate, performance, overall evaluation, and the relationship between the facilitator and the students and school teachers.

#### Secondary outcomes

##### Substance use and prevention-related skills promoted by the program

European Drug Addiction Prevention Questionnaire (Eu-Dap). This instrument gathers information, knowledge, history of consumption, and opinions about the consumption of substances of abuse, with a primary emphasis on alcohol, tobacco, and cannabis. The protective and risk factors for substance abuse are measured using several scales. Among these factors are positive and negative beliefs about substance use, expectations of future use, perceived risk of substance abuse, normative beliefs about substance use, school bonding, peer pressure, and decision-making skills, among others. For more details, see the validation for the Chilean adolescent population [42]. The Eu-Dap questionnaire was administered before (beginning of the academic year) and after the intervention (end of the academic year) at equivalent times for the control and the intervention groups.

### Data analysis

#### Primary outcomes

##### Acceptability

We described the frequencies of the degree of agreement with several statements by students regarding acceptability in the following aspects: general and implementation acceptability, usefulness, and satisfaction.

Additionally, we extracted qualitative data from the students´ surveys in relationship to the positive and negative perceptions of content, pedagogy, and logistics of the program, following the recommendations by Macklem [43]. Content refers to the knowledge, attitudes, values, norms, and skills addressed by the program and will be the mediators of the behavior change we want to prevent. Pedagogy involves the teaching methods, strategies, and facilitator-student interactions, which contribute to the effectiveness of the program. This component has also been called the “quality of delivery” of the program or how the facilitator implements the program (for example, the facilitator’s training, skills in the methods described in the programs, enthusiasm, preparation, attitudes, etc.). The logistics include the number of sessions, duration of each session, and frequency of sessions. We reported the frequencies revealed by the students in each of these categories.

##### Feasibility

We presented the number of schools and students participating at each study stage, the response rate for baseline and after-intervention surveys, and the time allocated to surveys and YSLQQ sessions.

##### Fidelity and quality of the implementation

We presented the proportion of sessions where facilitators and observers reported completing or conducting particular aspects of the implementation of the program, organized in six sections: fidelity of the program according to the manual, session times, classroom, performance, facilitator´s general evaluation, and the relationship between facilitator, school teacher, and schools.

#### Secondary outcomes

Firstly, we used descriptive statistics to assess balance across arms at baseline. The primary between-group analysis was carried out on an intention-to-treat basis for post-intervention monthly tobacco, alcohol, drunkenness, and cannabis prevalence and Eu-Dap subscales scores. We used linear mixed-effects model analysis to compare the intervention and control groups regarding the change in secondary outcome measures from baseline to post-intervention. Schools were included as a random factor for each outcome measure to account for possible school effects.

## Results

### Demographic data

**Fig. 1:**
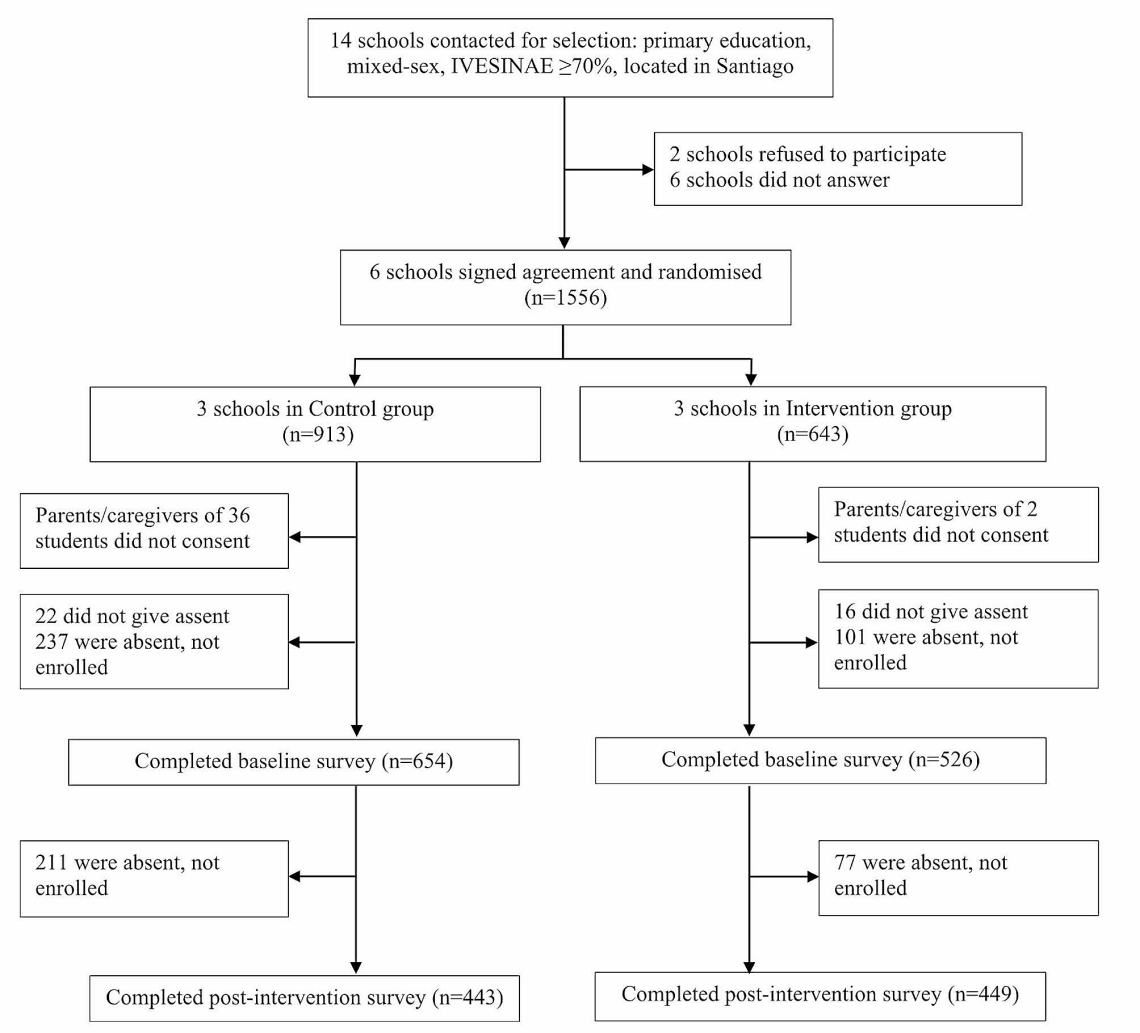
Flowchart

The total number of participants was 1180, divided into two groups: intervention (*n* = 526) and control (*n* = 654). The gender distribution was 637 males (53.9%) and 543 females (46.0%). The average age in both groups was 12 years old. At baseline, lifetime, 12-month, and last 30-day substance use prevalence was higher in the control group than in the intervention group. Both groups had similar Eu-Dap risk and protective factors subscale scores (see Table 1). For details on the number of students included at each stage and finally analyzed, see Fig. 1, Flowchart.

**Table 1:** Demographic features, substance use, and Eu-Dap risk and protective factors at baseline

Variable	Control		Intervention	
	*n*	%	*n*	%
Grade				
6th	212	32.4	158	30.0
7th	225	34.4	177	33.7
8th	217	33.2	191	36.3
Total	654		526	
Gender				
Male	338	51.7	299	56.8
Female	316	48.3	227	43.2
Eu-Dap Questionnaire prevalence				
Lifetime substance use				
Tobacco	107	16.4	63	12.2
Alcohol	306	47.4	197	38.0
Drunkenness	66	10.2	44	8.5
Cannabis	61	9.7	40	8.1
Last 12 months substance use				
Tobacco	53	8.2	27	5.2
Alcohol	167	25.7	93	18.0
Drunkenness	31	4.8	23	4.4
Cannabis	40	6.2	18	3.5
Last month substance use				
Tobacco	27	4.2	12	2.5
Alcohol	77	11.9	50	9.6
Drunkenness	19	2.9	12	2.3
Cannabis	27	4.2	11	2.1
	Mean	(SD)	Mean	(SD)
Age	12.4	1.1	12.3	1.0
Eu-Dap Risk and protective subscales				
Positive beliefs on Tobacco	11.1	4.3	11.4	4.7
Negative beliefs on Tobacco	13.0	4.3	13.0	4.4
Positive beliefs on Alcohol	11.0	4.7	11.2	4.8
Negative beliefs on Alcohol	16.3	5.7	16.7	5.9
Positive beliefs on Cannabis	12.1	5.3	12.2	5.5
Negative beliefs on Cannabis	17.3	6.0	17.6	6.1
Expectations of future drug use	7.9	3.6	7.9	3.7
Drug Use Index	0.1	0.9	0.2	1.1
Poor problem-solving skills	8.7	2.9	8.6	3.1
School bonding	11.8	2.7	12.2	2.5
Positive attitudes towards drug use	6.1	2.6	5.7	2.4
Negative attitudes towards drug use	21.2	5.3	20.7	6.0
Positive self-esteem	15.6	3.4	15.7	3.3
Assertive skills	18.5	3.6	18.5	3.5
Normative beliefs	2.9	3.9	2.3	3.8
Negative influence by friends	6.3	2.7	5.8	2.6

### Primary outcome

#### Acceptability

Regarding general acceptability, 49.6% of students liked the sessions, 52.1% liked the type of activities used in the sessions, 79.2% learned about the dangers of substances, and 65.8% thought that the program helped them develop more skills to avoid using tobacco, alcohol, or substances in the future; 62.9% were happy or very happy with the program in general. See more details in Table 2.

**Table 2:** Acceptability by students

	Strongly disagree		Disagree		Not agree nor disagree		Agree		Strongly agree	
General Acceptability	*n*	%	*n*	%	*n*	%	*n*	%	*n*	%
I liked the sessions	32.0	7.5	37.0	8.7	145.0	34.2	119.0	28.1	91.0	21.5
I learned	20.0	4.7	45.0	10.6	129.0	30.5	140.0	33.1	89.0	21.0
I liked the activities	42.0	9.9	39.0	9.2	122.0	28.8	123.0	29.0	98.0	23.1
I think the program helped me	34.0	8.0	50.0	11.8	128.0	30.2	130.0	30.7	82.0	19.3
Implementation Acceptability										
Teacher and Facilitator get along	11.0	2.6	3.0	0.7	68.0	16.1	140.0	33.1	201.0	47.5
Teacher participates and collaborates during the sessions	28.0	6.6	37.0	8.7	118.0	27.8	144.0	34.0	97.0	22.9
I like the type of activities used in the sessions	35.0	8.3	35.0	8.3	112.0	26.4	126.0	29.7	116.0	27.4
The booklet used helps me in during the sessions	36.0	8.5	50.0	11.8	124.0	29.2	137.0	32.3	77.0	18.2
I like the design and format of the booklet	49.0	11.6	41.0	9.7	132.0	31.1	121.0	28.5	81.0	19.1
Usefulness: “The program helped…										
… to improve school coexistence	20.0	4.7	33.0	7.8	124.0	29.2	163.0	38.4	84.0	19.8
… me learn how to relate to others better.	30.0	7.1	51.0	12.0	137.0	32.3	136.0	32.1	70.0	16.5
… me manage my emotions better	56.0	13.2	48.0	11.3	146.0	34.4	107.0	25.2	67.0	15.8
… to learn about the dangers of drugs.	16.0	3.8	12.0	2.8	60.0	14.2	155.0	36.6	181.0	42.7
… all classmates to relate better with each other.	32.0	7.5	46.0	10.8	148.0	34.9	138.0	32.5	60.0	14.2
… me **today** to have more skills to avoid using tobacco, alcohol or drugs	37.0	8.7	22.0	5.2	86.0	20.3	125.0	29.5	154.0	36.3
…me to have more skills to avoid using tobacco, alcohol, or drugs **in the future**	30.0	7.1	20.0	4.7	85.0	20.0	112.0	26.4	177.0	41.7
Satisfaction: “Thinking about the “YSLQQ” Program in general,…	Very unhappy		Unhappy		Nor happy nor unhappy		Happy		Very happy	
… how happy or satisfied are you with the facilitator?	15.0	3.5	9.0	2.1	84.0	19.8	149.0	35.1	167.0	39.4
… how happy or satisfied are you with the length of the program?	36.0	8.5	24.0	5.7	105.0	24.8	160.0	37.7	99.0	23.3
… how happy or satisfied are you with the activities?	34.0	8.0	27.0	6.4	110.0	25.9	143.0	33.7	110.0	25.9
… how happy or satisfied are you with the program in general?	25.0	5.9	25.0	5.9	107.0	25.3	146.0	34.5	120.0	28.4

When we asked about the positive aspects of the intervention, most comments were related to the use of pedagogical strategies (e.g., “We can do fun things like the activity of *emotions*”). Regarding the negative aspects, very few students were critics of the content. However, they mentioned that some sessions were long. See Table 3.

**Table 3:** Positive and negative perceptions about the program

Aspects of the program	Positive^1^			Negative^1^		
	*n*	%	Qualitative Example	*n*	%	Qualitative Example
Content	107	25.2	“It helps us to know about drugs and marijuana use”	11	2.6	“The activities were good but what I don’t like is that they are very repetitive”
Pedagogical methods	231	54.5	“What I liked the most is that we can do fun things like the activity of *emotions*”	188	44.3	“Unentertaining activities”
Logistics	0	0.0	No comments	77	18.2	“Long sessions”
General positive appreciation	16	3.8	“I liked the whole program because they are entertaining”	20	4.7	“I didn’t like it at all because I’m not interested in the program”
No response	70	16.5	No comments	128	30.2	No comments
Total^2^	424	100.0		424	100.0	

Note: *n* = number of students who wrote comments about the perception of the program on the Acceptability survey. ^1^ Both “Positive” and “Negative” columns should be read as independent because students in the Acceptability survey were able to mention either positive or negative aspects of the intervention. The responses were organized into three categories: Content, Pedagogy, and Logistics. ^2^ Total refers to the total number of students who completed the Acceptability survey

#### Feasibility

126 schools fulfill the inclusion criteria in the registry of the Ministry of Education in the Province of Santiago. Fourteen were contacted via email and phone calls. Two refused to participate, and six did not respond to emails or phone calls. Finally, six schools agreed to participate, which were randomly allocated to the control and intervention groups. In the control group, from 913 enrolled students, 654 completed the baseline questionnaire (71.6%), and then 443 students completed the post-intervention survey (48.5%). In the intervention group, of 643 enrolled students, 526 completed the baseline questionnaire (81.8%), and then 449 students completed the post-intervention survey (77.6%). In most occasions, baseline and post-intervention surveys were implemented in 45 min. However, in two schools, due to some important degree of student absence on the day of the survey, we scheduled another day during the same week to obtain more responses. The implementation of the session was planned to be delivered in 45 min., which was fulfilled on the great majority of occasions. However, even though some sessions needed to be rescheduled due to school issues (e.g., class suspension of school trips or to prepare national anniversary activities), all sessions were delivered during the academic year.

#### Fidelity and quality of the implementation (suppl 4)

Out of 284 sessions conducted during this pilot study, 276 facilitators´ session reports were analyzed (see Suppl 4, Tables 1-6) along with observers´ report of 66 (23.2%) video sessions recorded. These reports comprise six sections: Fidelity of the program according to the manual (Suppl 4, Table 1), session times (Suppl 4, Table 2), classroom climate (Suppl 4, Table 3a-3b), performance (Suppl 4, Table 4a-b), Facilitator´s general evaluation (Suppl 4, Table 5), and the relationship between facilitator, school teacher and schools (Suppl 4, Table 6a-6b).

**Table 4:** Secondary outcomes: substance use and Eu-dap risk and protective factors after intervention

Variable	Control % (95%CI)		Intervention % (95%CI)		OR (95% CI)*	*p*-value
	Pre	Post	Pre	Post		
Lifetime substance use						
Tobacco	16.4 (13.7–19.4)	17.2 (14.0–21.0)	12.2 (9.6–15.3)	15.6 (12.5–19.3)	0.92 (0.62–1.37)	0.681
Alcohol	47.4 (43.6–51.3)	4.3 (3.8–4.7)	38.0 (33.9–42.3)	4.1 (3.7–4.6)	1.04 (0.63–1.70)	0.880
Drunkenness	10.2 (8.1–12.8)	9.8 (7.3–12.9)	8.5 (6.4–11.2)	11.9 (9.2–13.2)	1.26 (0.72–2.22)	0.421
Cannabis	9.7 (7.7–12.3)	10.7 (8.1–14.0)	8.1 (6.0-10.9)	9.1 (6.7–12.3)	0.49 (0.23–1.05)	0.066
Last 12 months substance use						
Tobacco	8.2 (6.3–10.5)	7.7 (5.5–10.6)	5.2 (3.6–7.5)	8.3 (6.1 (11.3)	0.94 (0.68–1.29)	0.685
Alcohol	25.7 (22.5–29.2)	25.9 (22.2–30.2)	18.0 (14.9–21.5)	24.5 (20.7–28.7)	1.05 (0.56–1.95)	0.886
Drunkenness	4.8 (3.4–6.7)	**5.7 (3.9–8.3)**	4.4 (3.0-6.6)	**5.6 (3.8–8.2)**	**0.80 (0.69–0.93)**	**0.003**
Cannabis	6.2 (4.6–8.3)	8.7 (6.4–11.7)	3.5 (2.2–5.5)	7.2 (5.2–10.0)	0.50 (0.17–1.45)	0.204
Last 30-day substance use						
Tobacco	4.2 (2.9-6.0)	17.2 (14.0–21.0)	2.5 (1.5–4.3)	15.6 (12.4–19.3)	0.95 (0.67–1.35)	0.789
Alcohol	11.9 (9.6–14.6)	12.5 (10.0-15.9)	9.6 (7.4–12.5)	12.9 (10.1–16.3)	1.03 (0.61–1.74)	0.920
Drunkenness	2.9 (1.9–4.6)	**4.1 (2.6–6.4)**	2.3 (1.3-4.0)	**2.5 (1.4–4.4)**	**0.39 (0.21–0.72)**	**0.002**
Cannabis	4.2 (2.9-6.0)	**6.2 (4.3–8.9)**	2.1 1.2–3.8)	**3.6 (2.2–5.8)**	**0.42 (0.21–0.83)**	**0.013**
Eu-Dap Risk and protective subscales		**Mean (SD)**		**Mean (SD)**	β (95% CI)*	**p-value**
Positive beliefs on Tobacco	11.1 (4.3)	11.8 (4.5)	11.4 (4.7)	11.9 (4.8)	0.00 (-0.86 to 0.87)	0.996
Negative beliefs on Tobacco	13.0 (4.3)	14.0 (4.5)	13.0 (4.4)	13.6 (4.4)	-0.28 (-1.76 to 1.20)	0.647
Positive beliefs on Alcohol	11.0 (4.7)	11.5 (4.9)	11.2 (4.8)	11.6 (5.1)	0.13 (-0.47 to 0.73)	0.603
Negative beliefs on Alcohol	16.3 (5.7)	17.3 (5.7)	16.7 (5.9)	16.9 (6.0)	0.10 (-1.73 to 1.76)	0.988
Positive beliefs on Cannabis	12.1 (5.3)	12.5 (5.3)	12.2 (5.5)	12.5 (5.6)	0.13 (-0.52 to 0.78)	0.624
Negative beliefs on Cannabis	17.3 (6.0)	18.2 (5.8)	17.6 (6.1)	17.9 (6.0)	-0.11 (-1.55 to 1–32)	0.850
Expectations of future drug use	7.9 (3.6)	8.1 (3.8)	7.9 (3.7)	8.3 (4.1)	0.11 (-0.73 to 0.94)	0.760
Drug Use Index	0.1 (0.9)	**0.4 (1.5)**	0.2 (1.1)	**0.2 (1.1)**	**-0.30 (-0.45 to -0.15)**	**0.004**
Poor problem-solving skills	8.7 (2.9)	8.9 (2.3)	8.6 (3.1)	9.0 (3.0)	0.14 (-0.66 to 0.95)	0.665
School bonding	11.8 (2.7)	11.5 (2.6)	12.2 (2.5)	12.0 (2.5)	-0.01 (-1.00 to 0.97)	0.970
Positive attitudes towards drug use	6.1 (2.6)	6.6 (3.0)	5.7 (2.4)	6.1 (2.7)	-0.54 (-1.75 to 0.66)	0.297
Negative attitudes towards drug use	21.2 (5.3)	21.5 (11.4)	20.7 (6.0)	20.9 (6.0)	0.51 (-3.23 to 4.25)	0.740
Positive self-esteem	15.6 (3.4)	15.1 (3.7)	15.7 (3.3)	15.4 (3.3)	-0.04 (-0.97 to 0.89)	0.919
Assertive skills	18.5 (3.6)	18.1 (3.7)	18.5 (3.5)	18.3 (3.6)	-0.11 (-1.02 to 0.80)	0.764
Normative beliefs	2.9 (3.9)	3.6 (3.7)	2.3 (3.8)	3.0 (4.0)	-0.57 (-1.90 to 0.75)	0.318
Negative influence by friends	6.3 (2.7)	6.4 (2.9)	5.8 (2.6)	6.3 (2.7)	-0.09 (-0.37 to 0.20)	0.474

##### Program fidelity

Regarding implementing the 12 sessions, all classes received 100% of the sessions. In general, facilitators´ reports stated that several parts of the sessions were implemented according to the manual in more than 73.9% of sessions (see Suppl 4, Table 1), and in the report from the observer, this percentage was lower (25.8%).

##### Session times

Sessions were implemented in the allocated time or using less time in 90% of sessions, according to facilitators, and 89.4%, according to observers. (see Suppl 4, Table 2)

##### Climate in the classroom

Conflict was reported in 31.9% of the sessions by facilitators and 62.1% by observers. It was resolved on most occasions. Facilitators and observers responded that facilitators prepared the classroom space to deliver the session on most occasions. Regarding using strategies to capture students´ attention, facilitators said that in 86.6% of sessions, they always or almost always used these; however, observers reported that 55% of sessions never or almost never perceived the use of these strategies. In addition, the observers noted that the student’s level of attention and participation was high or very high in 69.6% of sessions. The level of participation in the activities was high or very high in 70.4% of sessions. Additionally, the degree of involvement of the school teacher in 51.4% of sessions was high or very high. (see Suppl 4, Table 3a-3b)

##### Performance

The Facilitator reported that in 87% of sessions, they had achieved a high level of knowledge (“I studied [the content of the session] and knew it perfectly”) that fit the program. In 81.2% of sessions, facilitators had no external interruptions that prevented the session from taking place smoothly. The necessary materials for developing the session were available in 90.9% of the sessions reported by facilitators and in 92.4% of sessions reported by observers. The facilitator demonstrated mastery of the intervention in 65.4% of sessions, delivering the contents with fluidity to complete the session. This perception was up to 98.5% in the case of observers. (see Suppl 4, Table 4a-4b)

##### Overall rating

In 77.8% of sessions, facilitators rated the overall evaluation of the session as 4 or 5. Regarding the positive and negative aspects, facilitators mentioned that student participation was a negative aspect in 39.7% of sessions, but at the same time, they highlighted the students´ attitudes as a positive aspect of the session.

##### Relationship between facilitators and school teachers/schools

In 96.7% of sessions, facilitators did not report any difficulties in the timetable and access to the school by the school authorities. The relationship with school teachers was positive in most sessions and collaborative in 73.6% of sessions (See Suppl 4, Table 6a). Observers reported that the school teachers were present in most sessions (84.8%), and facilitators encouraged their participation in 48.5% of sessions (See Suppl 4, Table 6b).

### Secondary outcomes

Regarding substance use, we found no differences between control and intervention groups in the lifetime prevalence of all substances measured. Regarding the last 12-month prevalence, drunkenness was significantly reduced (OR = 0.80, 95%CI: 0.69–0.93, *p* = 0.003) among students in the intervention group. In this time frame, there was also a reduction in cannabis use in the intervention group, but not significant. Finally, in the last 30-day prevalence, there was a significant reduction in drunkenness (OR = 0.39, 95%CI: 0.21–0.72, *p* = 0.002) and cannabis use (OR = 0.42, 95%CI: 0.21–0.83, *p* = 0.013) among students in the intervention group. See Table 4.

In the analysis of the effect of the intervention on changing the risk and protective factors measured by the Eu-Dap questionnaire, we only found a significant reduction in the Drug Abuse Index (*p* = 0.004) among students in the intervention group. This index refers to the presence of self-reported harm consequences after using alcohol or substances. See Table 4.

## Discussion

This is the first study exploring the acceptability, feasibility, and fidelity of implementing the program Unplugged, under the name of “Yo Sé Lo Que Quiero” (YSLQQ) among Spanish-speaking countries in Latin America. The intervention was well received by students, who mentioned that, generally, they liked the activities of sessions, they felt that they learned about the risk of using substances of abuse, and they thought that the skills promoted by the program would reduce the risks of using substances in the future. All sessions were implemented, and the facilitators reported high levels of fidelity and quality of the intervention. When reports from facilitators were compared with the observers of recorded sessions, there was a less optimal evaluation of the implementation of quality. The latter has also been found in other studies [44, 45].

This study also explored the efficacy of the intervention in reducing substance use and positively changing the risk and protective factors among students in the intervention group. These results showed that the YSLQQ program reduced the episodes of drunkenness and cannabis use, especially in the period of 30-day prevalence.

The Unplugged program has been tested in Europe [19, 37, 38] and Africa [26] with good results. Before our study was implemented, Unplugged was tested in Brazil, and the effectiveness of the two adaptations had mixed results [27, 29, 31]. In the first adaptation, the authors mentioned that the bad results were, in part, due to changes in the content of some sessions and problems in the fidelity of the implementation [27, 29]. In the second adaptation, with minimal and superficial changes in the program’s content, compared with the original Unplugged, program #Tamojunto 2.0 reduced alcohol initiation but appeared not to reduce past-month binge drinking among 8th -grade students in Brazil. All these results highlight the importance of three key elements: (1) conducting local evaluations of preventive programs that proved to be effective elsewhere because not always the results are equivalents; (2) being careful of keeping the core components of the original program to ensure that the mechanisms for what the program works are in place when doing the adaptation; and (3) to ensure that the implementation of the program is done with fidelity and quality.

Very few school-based interventions to prevent substance use among adolescents have been successfully implemented worldwide. Some examples are the School Health and Alcohol Harm Reduction Project (SHAHRP) in Australia [46], Life Skills Training (LST) program in the USA [23, 47, 48] and Unplugged in seven European countries [24]. Except for Unplugged, the other programs have not been studied in Latin America. Additionally, a recent randomized controlled trial testing the effectiveness of the Drug and Violence Resistance Educational Program (PROERD), which is the adapted and translated version of the North American program DARE-Keepin’it REAL. The results found no evidence of the effectiveness of PROERD as an intervention for the prevention of substance use, and the authors discussed several hypotheses for these results, such as poor adaptation, cultural differences between Brazil and the USA, and lack of implementation fidelity [49]. In the process of adapting Unplugged, our team worked closely with the Unplugged European Team to receive feedback and ensure that the adaptation proposed did not change the core components of the programs. Additionally, we carried out a system of close supervision and evaluation of the fidelity of the implementation, considering that adherence and dosage are important considerations concerning the effectiveness of the program [50].

It is important to consider the differential effect of these preventive programs on specific substances. The effectiveness of Unplugged in Europe has not had the same impact on tobacco, alcohol, or cannabis. For instance, the study conducted in the Czech Republic among 6th graders has presented good results on tobacco [51] and cannabis use [51, 52], but the results are not so good for alcohol [52]. In our study, we have found good results for drunkenness and cannabis use but no impact on tobacco use. The differential effect of preventive programs may be related to cultural differences. For example, in the Czech Republic, the social acceptance of heavy alcohol consumption seems to be high, making it more challenging to reach adolescents with a preventive message and generate behavioral changes with the implementation of this kind of program for one year [52]. In the same sense, Chile has been known to have the highest prevalence of tobacco use among adolescents in the Americas [16], making it plausible that the social acceptance of tobacco use is high. The mediating study of the Unplugged intervention also found that the different mediators have a differential role in specific substances [25]. Therefore, future research needs to consider these cultural differences and the differential role of mediators to make potential adjustments to the content of the program.

Regarding feasibility, our team was able to implement all 12 sessions in the intervention group. Considering other experiences [53, 54] where school teachers were trained to implement this kind of program with poor results in fidelity and implementation, we decided to train and supervise external facilitators. We consider this a successful implementation, and a larger RCT could continue this implementation to ensure adherence and fidelity. One of the difficulties we found was the higher rate of attrition found in the control group, possibly explained by the need for more incentives for participation due to not obtaining the intervention. Future evaluations must consider these results at the moment of recruitment to avoid problems of statistical power at the end of the assessments.

Finally, few studies have explored whether gender or age are potential modifiers of the effect of preventive programs on substance use. For instance, Vigna-Taglianti et al. found a significant association between participating in the Unplugged program and a lower prevalence of all behavioral outcomes among boys but not among girls [55]. Additionally, in the Czech Republic, Novak et al. (2013) [56] found that Unplugged intervention was associated with the reduction of any drunkenness among boys and any tobacco use among girls in the 30 days before testing. Furthermore, participating in the program was found to have positive effects on both genders’ cannabis use, with girls showing lower levels of cannabis use even 33 months after the baseline test. On the other hand, in a meta-analysis, programs targeting older, high school-aged youth (i.e., 14 years and older) were more effective than those targeting younger students (i.e., under 14 years) [54]. In the original evaluation of Unplugged in seven European countries, there were no references to the effectiveness of the program according to the grades of students where it was implemented. However, in the evaluation of Unplugged in the Czech Republic, the good results seem to apply only to 11-12-year-old kids [52]. Therefore, future research on this intervention in Chile may include the study of age differences and its preventive effect.

### Limitations

This was a small pilot study with no intention to provide strong evidence of the effectiveness of the intervention and the positive results on reducing drunkenness and cannabis use should be considered as preliminary evidence. It is advisable to wait for the results of a larger RCT before considering investing in scaling up this intervention in Chile or other Spanish-speaking Latin American countries. Within the limitations, it was possible to determine that the main evaluations were based on the self-reports of the participants, which may have a social desirability bias. During the execution of this intervention, Chile was affected by a social outburst. This scenario prevented the completion of the evaluation of some classes, impacting the trial in different ways for the control and intervention groups. For instance, there was less participation in the post-intervention assessment overall but at a higher rate in the control group, even though some classes in the intervention group could not be assessed post-intervention. The evaluation of the schools in the intervention group was scheduled for after the completion of the program; however, the intervention was delayed in some schools, and at the end of the academic year, schools were prematurely closed due to political violence. On the other hand, there was higher absenteeism for baseline and post-intervention assessment in the control group. Overall, there was less participation in the control group.

The schools in each group did not have the same size and administrative dependence characteristics, which occurred by chance. Given that the main objectives of this study were related to acceptability and feasibility, the results are very useful for future and larger evaluation of this intervention. One final commentary is related to the choice made by the research team to use external facilitators instead of school teachers to implement the program. It is well known the high workload of school teachers in their day-to-day activities [57] and the degree of absenteeism of teachers due to mental and physical problems in the Chilean educational system, especially after the COVID pandemic [58, 59]. These reasons motivated us to ask school teachers for help with disciplinary issues and collaborate with implementing activities when possible. Still, we rely on external facilitators to ensure the implementation, fidelity, and continuity of the program. Regarding sustainability and long-term program integration within schools, the participation of school teachers could be beneficial; however, given the current work conditions of teachers in Chile, it is challenging to consider this a real possibility for the future. In addition, the SENDA has created regional agencies in all Regions of Chile called “Previene” Agencies [60], where trained health professionals can assist schools with preventive actions. If a future larger RCT provides evidence of the effectiveness of this program, these SENDA professionals could be trained and be responsible for the implementation of the program in the schools.

## Conclusion

“Yo Sé Lo Que Quiero”, the Chilean version of the Unplugged substance use preventive program, seems to be well accepted by students and facilitators. Additionally, using external facilitators ensured good fidelity of the content of the program. Finally, the exploratory results of reducing drunkenness and cannabis use are promising to test the effectiveness of this intervention in larger RCTs in the future.

### Electronic supplementary material

Below is the link to the electronic supplementary material.

Supplementary Material 1

Supplementary Material 2

Supplementary Material 3

Supplementary Material 4

## Data Availability

The data that support the findings of this study are not openly available due to reasons of sensitivity and are available from the corresponding author upon reasonable request.
