# Supplementary material for: Acceptability, feasibility and fidelity of the culturally adapted version of Unplugged (“Yo Se Lo Que Quiero”), a substance use preventive program among adolescents in Chile: a pilot randomized controlled study
Source: BMC Public Health. 2024 Jul 29;24:2026. doi: 10.1186/s12889-024-19499-2 (PMC11285342; doi:10.1186/s12889-024-19499-2)
Supplement: Supplementary file 1 — Supplementary Material 1 [file 12889_2024_19499_MOESM1_ESM.docx]

Supplement 1: Brief description of YSLQQ sessions.

| Session | Name | Objectives | Focus | CASEL skills |
| --- | --- | --- | --- | --- |
| 1 | What is YSLQQ? | Introduction to the program, determination of the rules of the sessions, reflection on knowledge about drugs. | Information and attitudes | Self-awareness |
| 2 | Inside or outside the student group | Clarification of the influences of the student group and the expectations of the student group. | Interpersonal Skills | Social Awareness |
| 3 | Alcohol: Risks and Protection | Knowledge of the different factors that influence drug use. | Information and attitudes | Decision-making |
| 4 | Will it be as you think? | Critical evaluation of information, reflection on the differences between our opinion and the real data, correction of the rules. | Intrapersonal skills | Decision-making |
| 5 | What to expect from tobacco? | Information on the effects of smoking, differentiation between predicted and actual effects, as well as short-term and long-term effects. | Information and attitudes | Decision-making |
| 6 | With your own voice | Proper communication of emotions, distinction between verbal and non-verbal communication. | Intrapersonal skills | Self-awareness |
| 7 | Assert Yourself in the Face of Pressure | Promotion of self-confidence and respect for people. | Interpersonal Skills | Relational skills |
| 8 | On stage! | Giving and receiving compliments, practice, and reflection on making contact with others. | Intrapersonal skills | Relational skills |
| 9 | Know more, risk less | Information on the Effects of Drug Use | Information and attitudes | Decision-making |
| 10 | Strategies to face difficulties | Expressing negative feelings, coping with difficulties. | Intrapersonal skills | Self-awareness |
| 11 | Solve problems and face decisions | Structured problem solving, encouraging creative thinking and self-control. | Intrapersonal skills | Decision-making |
| 12 | Setting goals | Distinction between short-term and long-term goals. Assessment of the program. | Intrapersonal skills | Self-awareness |
