# Supplementary material for: Acceptability, feasibility and fidelity of the culturally adapted version of Unplugged (“Yo Se Lo Que Quiero”), a substance use preventive program among adolescents in Chile: a pilot randomized controlled study
Source: BMC Public Health. 2024 Jul 29;24:2026. doi: 10.1186/s12889-024-19499-2 (PMC11285342; doi:10.1186/s12889-024-19499-2)
Supplement: Supplementary file 2 — Supplementary Material 2 [file 12889_2024_19499_MOESM2_ESM.pdf]

STUDENT ACCEPTABILITY QUESTIONNAIRE
I Know What I Want Program Half

Student Name:____

School Name: ____

Course: __________________________________ Date: ____________________

Take a minute before answering these questions, there are no right answers, only personal opinions of what I experienced in the sessions of the I **Know What I Want Program**. It is very important for us to invite you to think about it and share it with us.

1. On a scale of 1 to 5, where 1 is "Strongly disagree" and 5 is "Strongly agree," indicate how you agree with the following sentences in **relation to what you experienced in the "I Know What I Want" program sessions**.

		**Strongly disagree**	**disagree**	**Not agree nor disagree**	**Agree**	**Strongly agree**
1	I liked the sessions	1	2	3	4	5
2	I learned a lot in the sessions	1	2	3	4	5
3	The activities we did in the Sessions were entertaining	1	2	3	4	5
4	I think the Program has given me helped	1	2	3	4	5

2. On the same scale as in the previous question, indicate how well you agree with the following sentences in **relation to the implementation of the "I Know What I Want" Program.**

		**Strongly disagree**	**Disagree**	**Not agree nor disagree**	**Agree**	**Strongly agree**
1	My school teacher and the teacher of the program get along well	1	2	3	4	5
2	My teacher at school participates and collaborates with the teacher of the program during the sessions	1	2	3	4	5
3	I like the type of activities used in sessions (e.g., games, making group activities, discussion)	1	2	3	4	5
4	The booklet used in the sessions helps to better understand what has been seen in the program	1	2	3	4	5
5	I like the design and format of the booklet	1	2	3	4	5

3. The "I Know What I Want" Program aims to deliver life skills, we would like to know your opinion about the effect of this program among the students of the course.

		Strongly disagree	Disagree	Not agree nor disagree	Agree	Strongly agree
1	This program is useful for **improving the school coexistence** in our course	1	2	3	4	5
2	This program has helped me learn how **to relate to others in a better** way	1	2	3	4	5
3	This program has helped me **manage better my emotions**	1	2	3	4	5
4	This program is useful for **learning about the dangers of drugs**	1	2	3	4	5
5	By participating in this program, I see that We all **learn to relate better**	1	2	3	4	5
6	I feel like **I have more skills** today to avoid using tobacco, alcohol, or drugs	1	2	3	4	5
7	I feel like I have more skills to avoid using tobacco, alcohol, or drugs **in the future**	1	2	3	4	5

4. Thinking about the "I Know What I Want" Program in general, how happy or satisfied are you with...?

		Very Unhappy	Unhappy	Nor happy Nor unhappy	Happy	Very Happy
1	... the treatment received by the Professor of the program	1	2	3	4	5
2	... The length of the program	1	2	3	4	5
3	... The activities	1	2	3	4	5
4	... The program as a whole	1	2	3	4	5

5. Are you happy with the length of each session (45 minutes)?

1	No, because it's too long
2	No, because it's too short
3	Yes, that's okay

6. What did you like most about the "I Know What I Want" Program? Indicate at least one aspect.

7. What did you like least about the "I Know What I Want" Program? Indicate at least one aspect.

8. What would you change so far about the "I Know What I Want" Program?

THANK YOU SO MUCH FOR PARTICIPATIN

CUESTIONARIO DE ACEPTABILIDAD DE ESTUDIANTES
Mitad del Programa Yo Sé Lo Que Quiero

Nombre Estudiante: ______________________________

Nombre del Colegio: ______________________________

Curso : ______________ Fecha ______ | ______ | ______

Toma un minuto antes de responder estas preguntas, no hay respuestas correctas, solo opiniones personales de lo vivido en las sesiones del Programa **Yo Sé Lo Que Quiero**. Para nosotros es muy importante invitarte a pensar y compartirlas con nosotros.

1. En una escala de 1 a 5, donde 1 es "Muy en desacuerdo y 5 es "Muy de acuerdo", señala cuál es tu grado de acuerdo con las siguientes oraciones **en relación con lo que viviste en las sesiones del programa "Yo sé lo que quiero"**.

		**Muy en desacuerdo**	**En desacuerdo**	**Ni de acuerdo, ni en desacuerdo**	**De acuerdo**	**Muy de acuerdo**
1	Me gustaron las sesiones	1	2	3	4	5
2	Aprendí mucho en las sesiones	1	2	3	4	5
3	Las actividades que hicimos en las sesiones fueron entretenidas	1	2	3	4	5
4	Pienso que el Programa me ha ayudado	1	2	3	4	5

2. En la misma escala que en la pregunta anterior, señala cuál es tu grado de acuerdo con las siguientes oraciones en **relación con la implementación del Programa "Yo sé lo que quiero".**

		**Muy en desacuerdo**	**En desacuerdo**	**Ni de acuerdo, ni en desacuerdo**	**De acuerdo**	**Muy de acuerdo**
1	Mi profesor/a del colegio y la Profesora del programa se llevan bien	1	2	3	4	5
2	Mi profesor/a del colegio participa y colabora con la Profesora del programa durante las sesiones	1	2	3	4	5
3	Me gusta el tipo de actividades usada en las sesiones (por ejemplo, juegos, hacer actividades de grupo, actividades de discusión)	1	2	3	4	5
4	El cuadernillo usado en las sesiones me ayuda a entender mejor lo visto en el programa	1	2	3	4	5
5	Me gusta el diseño y formato del cuadernillo	1	2	3	4	5

3. El Programa "Yo sé lo que quiero" tiene como objetivo entregar habilidades para la vida, nos gustaría saber tu opinión sobre el efecto de este programa entre las y los estudiantes del curso.

		**Muy en desacuerdo**	**En desacuerdo**	**Ni de acuerdo, ni en desacuerdo**	**De acuerdo**	**Muy de acuerdo**
1	Este programa es útil para **mejorar la convivencia escolar** en nuestro curso.	1	2	3	4	5
2	Este programa me ha ayudado a aprender cómo **relacionarme con otros de mejor** manera.	1	2	3	4	5
3	Este programa me ha ayudado a **manejar mejor mis emociones**	1	2	3	4	5
4	Este programa es útil para **aprender sobre los peligros sobre las drogas**.	1	2	3	4	5
5	Al participar de este programa, veo que todos **aprendemos a relacionarnos mejor**.	1	2	3	4	5
6	Siento que **hoy** tengo más habilidades para evitar consumir tabaco, alcohol o drogas	1	2	3	4	5
7	Siento que tengo más habilidades para evitar consumir tabaco, alcohol o drogas **en el futuro**	1	2	3	4	5

4. Pensando en el Programa "Yo sé lo que quiero" en general, ¿cuán contento/a o satisfecho/a estás con…?

		**Muy insatisfecho/a**	**Insatisfecho/a**	**Ni satisfecho, ni insatisfecho/a**	**Satisfecho/a**	**Muy satisfecho/a**
1	…el trato recibido por la Profesora del programa	1	2	3	4	5
2	…la duración del programa	1	2	3	4	5
3	…las actividades	1	2	3	4	5
4	… el programa en general	1	2	3	4	5

5. ¿Estás contento/a con la duración de cada sesión (45 minutos)?

1	No, porque es muy larga
2	No, porque es muy corta
3	Sí, está bien

6. ¿Qué es lo que más te ha gustado del Programa "Yo sé lo que quiero" ? Indica al menos un aspecto.

7. ¿Qué es lo que menos te ha gustado del Programa "Yo sé lo que quiero" ? Indica al menos un aspecto.

8. ¿Qué cambiarías hasta ahora del Programa "Yo sé lo que quiero"?

¡MUCHAS GRACIAS POR PARTICIPAR!
