# Supplementary material for: Acceptability, feasibility and fidelity of the culturally adapted version of Unplugged (“Yo Se Lo Que Quiero”), a substance use preventive program among adolescents in Chile: a pilot randomized controlled study
Source: BMC Public Health. 2024 Jul 29;24:2026. doi: 10.1186/s12889-024-19499-2 (PMC11285342; doi:10.1186/s12889-024-19499-2)
Supplement: Supplementary file 3 — Supplementary Material 3 [file 12889_2024_19499_MOESM3_ESM.pdf]

“Yo Se Lo Que Quiero” Forms

*Obligatory

1. Facilitator *

Mark only one oval.

- Facilitator 1,
- Facilitator 2,
- Facilitator 3,
- Facilitator 4,
- Facilitator 5,
- Facilitator 6,
- Facilitator 7,

2. School

Mark only one oval.

- School 1
- School 2
- School 3

3. Grade

Mark only one oval.

- 6ºA
- 6ºB
- 6ºC
- 7ºA
- 7ºB
- 7ºC
- 8ºA
- 8ºB
- 8ºC

4. Session

Mark only one oval.

- 1
- 2
- 3
- 4
- 5
- 6
- 7
- 8
- 9
- 10
- 11
- 12

5. Date of application

____

Example: January 7, 2019

6. Report Type *

Mark only one oval.

( ) Session Report
( ) Video Observation *Skip to question 60*
( ) Live Session Observation

Session Report

7. Start time

____

Example: 8:30 a.m.

8. End Time

____

Example: 8:30 a.m.

9. Was the session recorded?

Mark only one oval.

( ) Yes
( ) No *Skip to question 12*

Untitled section

10. Video Length

11. Comments on the video

Fidelity section

Program fidelity according to manual

Next, indicate which of the following activities were carried out during the session under review

12. Start: Recap of the previous session

Mark only one oval.

- I didn't do the activity
- I did the activity but NOT according to the manual
- I performed the activity according to the manual

13. Start: Presentation of the objective

Mark only one oval.

- I didn't do the activity
- I did the activity but NOT according to the manual
- I performed the activity according to the manual

14. Comment on the activities that were not performed, that were not performed according to the man, and those extras to the manual

Program fidelity according to manual

Development of activities planned per Session.

15. Aperture

Mark only one oval.

- I didn't do the activity
- I did the activity but NOT according to the manual
- I performed the activity according to the manual

16. Core Activity 1

Mark only one oval.

- I didn't do the activity
- I did the activity but NOT according to the manual
- I performed the activity according to the manual

17. Core Activity 2

Mark only one oval.

- I didn't do the activity
- I did the activity but NOT according to the manual
- I performed the activity according to the manual
- Not applicable

18. Core Activity 3

Mark only one oval.

- I didn't do the activity
- I did the activity but NOT according to the manual
- I performed the activity according to the manual
- Not applicable

19. Comment on the implementation of aperture

20. Comment on the implementation of activity 1

21. Comment on the implementation of activity 2

22. Comment on the implementation of activity 3

23. Closing: Final Thoughts

Mark only one oval.

- I didn't do the activity
- I did the activity but NOT according to the manual
- I performed the activity according to the manual
- Not applicable

24. Closing: Another Unplanned Activity

Mark only one oval.

- I didn't do the activity
- I did the activity but NOT according to the manual
- I performed the activity according to the manual
- Not applicable

25. Comment on activities that were not done during the closure, those that were not done according to the manual, and those that were not in the manual

____

____

____

____

____

Session Times

26. Start-up time

Mark only one oval.

- I Used Less Time Than Allotted
- I Met Allotted Time
- I Exceeded Allotted Time

27. Core Activities Time

Mark only one oval.

- I Used Less Time Than Allotted
- I Met Allotted Time
- I Exceeded Allotted Time

28. Closing Time

Mark only one oval.

- I Used Less Time Than Allotted
- I Met Allotted Time
- I Exceeded Allotted Time

29. Overall Session Time

Mark only one oval.

- I Used Less Time Than Allotted
- I Met Allotted Time
- I Exceeded Allotted Time

30. Comment on the activities where you spent less time than allotted

31. Comment on the activities in which you exceeded the allotted time

Classroom Climate

32. During the course of the session, were there any conflict situations?

Mark only one oval.

- Yes *Skip to question 33*
- No *Skip to question 37*

Untitled section

33. Describe

34. How many conflict situations arose during the Session under review?

35. Were all conflict situations resolved?

Mark only one oval.

- ◯ Yes
- ◯ No

36. In general, to resolve conflict situations, which of the following steps were used?

Select all that apply.

- ☐ Understanding Student Needs
- ☐ Empathizing with Students
- ☐ Express concern about the student's behavior and its consequences
- ☐ Resolve the conflict situation in a directive manner
- ☐ Resolve the conflict situation collaboratively
- ☐ Address the conflict, but pause its resolution, to take it up again later

Untitled section

37. Check if you address the following skills during the Assessed Session

Select all that apply.

- ☐ Self
- ☐ Social Awareness
- ☐ Decision Making
- ☐ Self-Knowledge
- ☐ Relational skills

In relation to maintaining attention and encouraging student participation.

38. I prepared the space for a proper development of the session

Mark only one oval.

	1	2	3	4	5	
Never	◯	◯	◯	◯	◯	Always

39. I facilitated the students' active participation during the session, using strategies to promote the participation of the majority of the students.

Mark only one oval.

	1	2	3	4	5	
Never	◯	◯	◯	◯	◯	Always

40. I used strategies to capture and keep students' attention.

Mark only one oval.

	1	2	3	4	5	
Never	◯	◯	◯	◯	◯	Always

For the following items, please indicate the level at which they were presented during the session.

41. Students' level of attention

Mark only one oval.

	1	2	3	4	5	
Very low	○	○	○	○	○	Very high

42. Level of student participation in activities

Mark only one oval.

	1	2	3	4	5	
Very low	○	○	○	○	○	Very high

43. Level of teacher involvement

Mark only one oval.

	1	2	3	4	5	
Very low	○	○	○	○	○	Very high

Performance

44. What level of knowledge about the session did you achieve in the development of it?

Mark only one oval.

- I didn't study it and I didn't know it
- I read it and had a general idea, but I needed support from the Handbook all the time
- I read it and I had a general idea, but I needed support from the Manual much of the time
- . I studied it and I knew it, but I needed support from the Manual
- I studied it and I knew it perfectly

45. Did you have external interruptions that prevented you from having a smooth session ? (e.g. preventive activity against earthquakes, rehearsals of school events, external noises, etc.)

Mark only one oval.

- Yes *Skip to question 46*
- No *Skip to question 47*

46. Describe the interruptions that occurred in the Session

Untitled section

47. Did you have the appropriate materials for the session?

Mark only one oval.

◯ It does not have all the necessary materials ◯ Partially has the necessary materials ◯ It has all the necessary materials

48. The facilitator demonstrates mastery of the contents of the session and therefore presents himself with fluidity

Mark only one oval.

◯ The session is interrupted by the facilitator, and it is impossible to resume the dialogue with the students to complete the session. (e.g., "I forgot," "It wasn't like that," "Where are the materials?").

◯ The session is carried out with interruptions on the part of the facilitator, however, with effort the dialogue with the students can be resumed to complete the session

◯ The session is interrupted by the facilitator, however, the dialogue with the students can be easily resumed to complete the session.

◯ The session is carried out without interruptions on the part of the facilitator, which allows the delivery of the contents with fluidity to complete the session (It can be supported by the manual, but without generating breaks in the dialogue with the students)

49. Regarding the promotion of the participation of all students. State what you did

Select all that apply.

☐ Use equitable participant selection strategies (e.g., name pool)

☐ It allows the participation of different students during the session by asking directive questions by giving you the opportunity to respond first to those who have not participated

☐ Use positive strategies to capture and/or regain students' attention (e.g., playful dynamics to regain attention such as active singing, phrases, and breaks)

Overall rating

50. On a scale where 1 is "Poor" and 5 is "Excellent", how would you rate the session overall?

Mark only one oval.

	1	2	3	4	5	
Suitcase	⬭	⬭	⬭	⬭	⬭	Excellent

51. What positive aspects of the Session would you highlight as a whole?

52. What aspects would you define as negative about the Session in general?

53. What suggestions would you make to the Session as a whole?

Relationship with the establishment

54. Was there any difficulty in the timetable and access to the school due to the permission of the school authorities?

Mark only one oval.

- Yes *Skip to question 55*
- No *Skip to question 56*

Untitled section

55. Which one? Please provide any comments

Untitled section

56. Did the teacher or the school put any obstacles in the way of the session?

Mark only one oval.

- Yes *Skip to question 57*
- No *Skip to question 58*

Untitled section

57. Which one? Please provide any comments

Untitled section

58. Does the teacher of the Establishment show interest in collaborating or participating in the Session?

Mark only one oval.

- Yes
- No

59. Please provide any comments

____

____

____

____

____

Video Observation

60. Facilitator who conducted the workshop

Mark only one oval.

- ⬭ Facilitator 1
- ⬭ Facilitator 2

1. Implementation Fidelity

61. 1.1.1 Beginning: Recap of the previous session

Mark only one oval.

- ⬭ The activity is not carried out
- ⬭ It is done but not according to the manual
- ⬭ It is done according to the manual

62. 1.1.2 Beginning: Presentation of the objective

Mark only one oval.

- The activity is not carried out
- It is done but not according to the manual
- It is done according to the manual

63. 1.2 Did you do an unplanned activity for this part of the session?

Mark only one oval.

- Yes
- No

64. 1.3 Make a comment regarding activities that were not performed, were not performed according to the manual, and other activities performed that were not in the manual

65. 1.4.1 Planned Development by Session: Opening

Mark only one oval.

- The activity is not carried out
- The activity is performed but not according to the manual
- The activity is performed according to the manual
- Not applicable

66. 1.4.2 Planned Development by Session: Core Activity 1

Mark only one oval.

- The activity is not carried out
- The activity is performed but not according to the manual
- The activity is performed according to the manual
- Not applicable

67. 1.4.3 Planned Development by Session: Core Activity 2

Mark only one oval.

- The activity is not carried out
- The activity is performed but not according to the manual
- The activity is performed according to the manual
- Not applicable

68. 1.4.4 Planned Development by Session: Core Activity 3

Mark only one oval.

- The activity is not carried out
- The activity is performed but not according to the manual
- The activity is performed according to the manual
- Not applicable

69. 1.5 Comment on the implementation of the appeyfiura

70. 1.6 Comment on the implementation of core activity 1

71. 1.7 Comment on the implementation of core activity 2

72. 1.8 Comment on the implementation of core activity 3

73. 1.9 Closing: Final Thoughts

Mark only one oval.

- The activity is not carried out
- The activity is performed but not according to the manual
- The activity is performed according to the manual

74. 1.10 Did you do any unplanned activities in this part of the session?

Mark only one oval.

- Yes
- No

75. 1.11 Comment on activities that were not performed, those that were performed in accordance with the manual, and other activities performed that were not within the manual

Session Time

76. 1.12.1 Startup Time

Mark only one oval.

- Used less than allotted time
- Meets allotted time
- Exceeds allotted time

77. 1.12.2 Core Activity Time

Mark only one oval.

- Used less than allotted time
- Meets allotted time
- Exceeds allotted time

78. 1.12.3 Closing Time

Mark only one oval.

- Used less than allotted time
- Meets allotted time
- Exceeds allotted time

79. 1.12.4 Overall Session Time

Mark only one oval.

- Used less than allotted time
- Meets allotted time
- Exceeds allotted time

80. 1.13 Make a comment about activities that used less time than allotted or exceeded the allotted time

Classroom Climate

81. 2.1 Were there any conflict situations during the session?

Mark only one oval.

- Yes
- No *Skip to question 85*

Untitled section

82. 2.2 How many conflict situations arose during the session assessed?

____

83. 2.3 Were all conflict situations resolved?

Mark only one oval.

- Yes
- No

84. 2.4 In general, to resolve conflict situations, which of the following steps were used?

Select all that apply.

- [ ] Understanding Students' Needs
- [ ] Empathizing with Students
- [ ] Express the Monitor's concern about the student's behavior and its consequences
- [ ] Resolve the conflict situation in a directive manner
- [ ] Resolve the conflict situation collaboratively
- [ ] Address the conflict, but pause its resolution, to take it up again later

Untitled section

85. 2.5 Check if you address the following skills during the assessed session

Select all that apply.

- [ ] Self
- [ ] Social Awareness
- [ ] Decision Making
- [ ] Self-Knowledge
- [ ] Relational skills

2.6 In relation to maintaining attention and encouraging student participation, the facilitator...

86. .... prepared the space for a proper development of the session

Mark only one oval.

	1	2	3	4	5	
Never	○	○	○	○	○	Always

87. ... He used strategies to capture and keep students' attention.

Mark only one oval.

	1	2	3	4	5	
Never	○	○	○	○	○	Always

88. 2.7 Thinking about the performance during the evaluated session, in which of the following teaching styles does the facilitator situate?

Mark only one oval.

- Authoritarian,
- Autotitative,
- Permissive,
- Negligent

Performance

89. 3.1 Does the facilitator have the appropriate session materials?

Mark only one oval.

- It does not have all the necessary materials
- Partially has the necessary materials
- It has all the necessary materials

90. 3.2 The facilitator demonstrates mastery of the contents of the session and therefore presents himself or herself as a
fluidity

Mark only one oval.

- The session is interrupted by the facilitator, and it is impossible to resume the dialogue with the students to complete the session. (e.g., "I forgot," "It wasn't like that," "Where are the materials?").
- The session is carried out with interruptions on the part of the facilitator, however, with effort the dialogue with the students can be resumed to complete the session
- The session is interrupted by the facilitator, however, the dialogue with the students can be easily resumed to complete the session.
- The session is carried out without interruptions on the part of the facilitator, which allows the delivery of the contents with fluidity to complete the session (It can be supported by the manual, but without generating breaks in the dialogue with the students)

91. 3.3 Encourage the participation of all students, indicate whether the facilitator:

Select all that apply.

- [ ] Use equitable participant selection strategies (e.g., name pool)
- [ ] It allows the participation of different students during the session by asking directive questions by giving you the opportunity to respond first to those who have not participated
- [ ] Use positive strategies to capture and/or regain students' attention (e.g., playful dynamics to regain attention such as active singing, phrases, and breaks)

4. Facilitator Relationships

92. 4.1 Maintain a respectful and warm relationship with students, indicate if the facilitator performs any of the following behaviors

Select all that apply.

- [ ] Active Listening
- [ ] Uses a cordial tone to refer to students
- [ ] Takes opinions and makes them part of the activity
- [ ] Says hello and goodbye to students
- [ ] Maintain a positive and friendly attitude

93. 4.2 Is attentive to the needs of the students during the session (Breaks if he/she observes the children are very tired/active, if necessary he/she addresses problems in the session or suspends it, etc.)

Mark only one oval.

- Not attentive to those needs
- Is attentive to needs, but does not attempt to incorporate students who have those needs
- He is attentive and incorporates them by promoting the participation of all students

94. 4.3 Is the teacher in the classroom during the course of the session?

Mark only one oval.

- Yes
- No

95. 4.4 If the teacher is in the room, does the facilitator encourage the teacher's participation during the course of the session? (e.g., asks you a direct question, asks you for help, offers to take an action, etc.).

Mark only one oval.

- Yes
- No

Formularios Yo sé lo que quiero

*Obligatorio

1. Facilitador *

Marca solo un óvalo.

- Facilitador 1
- Facilitador 2
- Facilitador 3
- Facilitador 4
- Facilitador 5
- Facilitador 6
- Facilitador 7

2. Colegio

Marca solo un óvalo.

- Colegio 1
- Colegio 2
- Colegio 3

3. Curso

Marca solo un óvalo.

- 6ºA
- 6ºB
- 6ºC
- 7ºA
- 7ºB
- 7ºC
- 8ºA
- 8ºB
- 8ºC

4. Sesión

Marca solo un óvalo.

- 1
- 2
- 3
- 4
- 5
- 6
- 7
- 8
- 9
- 10
- 11
- 12

5. Fecha aplicación

Ejemplo: 7 de enero del 2019

6. Tipo de reporte *

Marca solo un óvalo.

- Reporte de sesión
- Observación video *Salta a la pregunta 60*
- Observación sesión en vivo

Reporte de sesión

7. Hora inicio

Ejemplo: 8:30 a.m.

8. Hora término

Ejemplo: 8:30 a.m.

9. ¿La sesión fue grabada?

Marca solo un óvalo.

- Sí
- No *Salta a la pregunta 12*

Sección sin título

10. Duración del video

____

11. Comentarios sobre el video

____
____
____
____
____

Sección sin título

Fidelidad al programa según manual

A continuación señale cuál de las siguientes actividades se realizaron durante la sesión evaluada

12. Inicio: Recapitulación de la sesión anterior

Marca solo un óvalo.

- ( ) No realicé la actividad
- ( ) Realicé la actividad pero NO de acuerdo al manual
- ( ) Realicé la actividad de acuerdo al manual

13. Inicio: Presentación del objetivo

Marca solo un óvalo.

- ◯ No realicé la actividad
- ◯ Realicé la actividad pero NO de acuerdo al manual
- ◯ Realicé la actividad de acuerdo al manual

14. Comente sobre las actividades que no se realizaron, que no se realizaron de acuerdo al manu y aquellas extras al manual

____
____
____
____
____

Fidelidad al programa según manual

Desarrollo de actividades planificadas por Sesión.

15. Apertura

Marca solo un óvalo.

- ◯ No realicé la actividad
- ◯ Realicé la actividad pero NO de acuerdo al manual
- ◯ Realicé la actividad de acuerdo al manual

16. Actividad central 1

Marca solo un óvalo.

- No realicé la actividad
- Realicé la actividad pero NO de acuerdo al manual
- Realicé la actividad de acuerdo al manual

17. Actividad central 2

Marca solo un óvalo.

- No realicé la actividad
- Realicé la actividad pero NO de acuerdo al manual
- Realicé la actividad de acuerdo al manual
- No aplica

18. Actividad central 3

Marca solo un óvalo.

- No realicé la actividad
- Realicé la actividad pero NO de acuerdo al manual
- Realicé la actividad de acuerdo al manual
- No aplica

19. Haga un comentario sobre la implementación de la apertura

20. Haga un comentario sobre la implementación de la actividad 1

21. Haga un comentario sobre la implementación de la actividad 2

22. Haga un comentario sobre la implementación de la actividad 3

23. Cierre: Reflexión final

Marca solo un óvalo.

- No realicé la actividad
- Realicé la actividad pero NO de acuerdo al manual
- Realicé la actividad de acuerdo al manual
- No aplica

24. Cierre: Otra actividad no planificada

Marca solo un óvalo.

- No realicé la actividad
- Realicé la actividad pero NO de acuerdo al manual
- Realicé la actividad de acuerdo al manual
- No aplica

25. Realice un comentario sobre las actividades que no se realizaron durante el cierre, aquellas q
no se realizaron de acuerdo al manual y aquejas que no estaban en el manual

Tiempos de la sesión

26. Tiempo de inicio

Marca solo un óvalo.

- Usé menos tiempo del asignado
- Cumplí con el tiempo asignado
- Excedí el tiempo asignado

27. Tiempo de actividades centrales

Marca solo un óvalo.

- Usé menos tiempo del asignado
- Cumplí con el tiempo asignado
- Excedí el tiempo asignado

28. Tiempo de cierre

Marca solo un óvalo.

- Usé menos tiempo del asignado
- Cumplí con el tiempo asignado
- Excedí el tiempo asignado

29. Tiempo de la sesión en general

Marca solo un óvalo.

- Usé menos tiempo del asignado
- Cumplí con el tiempo asignado
- Excedí el tiempo asignado

30. Realice un comentario sobre las actividades en que usó menos tiempo del asignado

31. Realice un comentario sobre las actividades en que excedió el tiempo asignado

Clima del aula

32. En el desarrollo de la Sesión, ¿se presentaron situaciones de conflicto?

Marca solo un óvalo.

- Sí *Salta a la pregunta 33*
- No *Salta a la pregunta 37*

Sección sin título

33. Describa

34. ¿Cuántas situaciones de conflicto se presentaron durante la Sesión evaluada?

35. ¿Todas las situaciones de conflicto fueron resueltas?

Marca solo un óvalo.

- Sí
- No

36. En general, para resolver situaciones de conflicto, ¿cuáles de los siguientes pasos se utilizaro

Selecciona todos los que correspondan.

- [ ] Entender las necesidades de los estudiante
- [ ] Empatizar con los estudiantes
- [ ] Expresar la preocupación por la conducta del estudiante y sus consecuencias
- [ ] Resolver en forma directiva la situación de conflicto
- [ ] Resolver en forma colaborativa la situación de conflicto
- [ ] Abordar el conflicto, pero dejar en pausa su resolución, para retomarlo después

Sección sin título

37. Marque si aborda las siguientes habilidades durante la Sesión evaluada

Selecciona todos los que correspondan.

- [ ] Automanejo
- [ ] Conciencia social
- [ ] Toma de decisiones
- [ ] Autoconocimiento
- [ ] Habilidades relacionales

En relación con mantener la atención y favorecer la participación de los estudiantes.

38. Preparé el espacio para un adecuado desarrollo de la sesión

Marca solo un óvalo.

	1	2	3	4	5	
Nunca	⬭	⬭	⬭	⬭	⬭	Siempre

39. Facilité que los estudiantes participaran de manera activa durante la sesión, utilizando estrategias para promover la participación de la mayoría de los estudiantes.

Marca solo un óvalo.

	1	2	3	4	5	
Nunca	⬭	⬭	⬭	⬭	⬭	Siempre

40. Utilicé estrategias para captar y mantener la atención de los estudiantes.

Marca solo un óvalo.

	1	2	3	4	5	
Nunca	⬭	⬭	⬭	⬭	⬭	Siempre

Respecto de los siguientes elementos, indique el nivel en que se presentaron durante la sesión.

41. Nivel de atención de los estudiantes

Marca solo un óvalo.

	1	2	3	4	5	
Muy bajo	◯	◯	◯	◯	◯	Muy alto

42. Nivel de participación de los estudiantes en las actividades

Marca solo un óvalo.

	1	2	3	4	5	
Muy bajo	◯	◯	◯	◯	◯	Muy alto

43. Nivel de involucramiento del/la docente

Marca solo un óvalo.

	1	2	3	4	5	
Muy bajo	◯	◯	◯	◯	◯	Muy alto

Desempeño

44. ¿Qué nivel de conocimiento sobre la sesión alcanzaste en el desarrollo de ésta?

Marca solo un óvalo.

- No la estudié y no la sabía
- La leí y tenía una idea general, pero necesité apoyo del Manual todo el tiempo
- La leí y tenía una idea general, pero necesité apoyo del Manual gran parte del tiempo
- La estudié y me la sabía, pero necesité apoyo del Manual
- La estudié y me la sabía perfectamente

45. ¿Tuvo interrupciones externas que le impidieron tener una sesión fluida? (ej: actividad preventiva frente a sismos, ensayos de actos escolares, ruidos externos, etc.)

Marca solo un óvalo.

- Sí *Salta a la pregunta 46*
- No *Salta a la pregunta 47*

46. Describa las interrupciones sucedidas en la Sesión

____

____

____

____

____

Sección sin título

47. ¿Contó con los materiales correspondientes de la sesión?

Marca solo un óvalo.

- No cuenta con todos los materiales necesarios
- Cuenta parcialmente con los materiales necesarios
- Cuenta con todos los materiales necesarios

48. El facilitador demuestra manejo de los contenidos de la sesión y por tanto se presenta con fluidez

Marca solo un óvalo.

- La sesión se realiza con interrupciones de parte del facilitador, y es imposible retomar el diálogo con los estudiantes para completar la sesión. (ej: "Se me olvidó", "No era así", "¿Dónde están los materiales?").
- La sesión se realiza con interrupciones de parte del facilitador, sin embargo, con esfuerzo se pued retomar el diálogo con los estudiantes para completar la sesión
- La sesión se realiza con interrupciones de parte del facilitador, sin embargo, se puede retomar el dialogo fácilmente con los estudiantes para completar la sesión.
- La sesión se realiza sin interrupciones de parte del facilitador, lo cual permite la entrega de los contenidos con fluidez para completar la sesión (Puede apoyarse en el manual, pero sin generar quiebres en el diálogo con los estudiantes)

49. Respecto de la promoción de la participación de todos los estudiantes. Indique lo que realizó

Selecciona todos los que correspondan.

- [ ] Utiliza estrategias equitativas de selección de participantes (ej: bolsa de nombres)
- [ ] Permite la participación de diferentes estudiantes durante la sesión realizando preguntas directivas otorgándole la oportunidad de responder primero a aquellos que no han participado
- [ ] Utiliza estrategias positivas para captar y/o retomar la atención de los estudiantes (ej: dinámicas lúdicas para retomar la atención como cantos, frases y quiebres activos)

Evaluación General

50. En una escala donde 1 es "Mala" y 5 es "Excelente", ¿Cómo evaluaría la sesión en general?

Marca solo un óvalo.

	1	2	3	4	5	
Mala	⬭	⬭	⬭	⬭	⬭	Excelente

51. ¿Qué aspectos resaltaría como positivos de la Sesión en general?

52. ¿Qué aspectos definiría como negativos de la Sesión en general?

53. ¿Qué sugerencias haría a la Sesión en general?

Relación con el establecimiento

54. ¿Hubo alguna dificultad en el horario y acceso al colegio por parte de las autoridades del establecimiento?

Marca solo un óvalo.

- Sí *Salta a la pregunta 55*
- No *Salta a la pregunta 56*

Sección sin título

55. ¿Cuál? Indique algún comentario

Sección sin título

56. ¿El/la profesor/a o el establecimiento puso algún obstáculo para la realización de la sesión?

Marca solo un óvalo.

- Sí *Salta a la pregunta 57*
- No *Salta a la pregunta 58*

Sección sin título

57. ¿Cuál? Indique algún comentario

Sección sin título

58. ¿El/la profesor/a del Establecimiento muestra interés en colaborar o participar de la Sesión?

Marca solo un óvalo.

- Sí
- No

59. Indique algún comentario

____
____
____
____
____

Observación video

60. Facilitadora que realizó el taller

Marca solo un óvalo.

- ( ) Facilitadora 1
- ( ) Facilitadora 2

1. Fidelidad de la implementación

61. 1.1.1 Inicio: Recapitulación de la sesión anterior

Marca solo un óvalo.

- ( ) No se realiza la actividad
- ( ) Se realiza pero no de acuerdo al manual
- ( ) Se realiza de acuerdo al manual

62. 1.1.2 Inicio: Presentación del objetivo

Marca solo un óvalo.

- No se realiza la actividad
- Se realiza pero no de acuerdo al manual
- Se realiza de acuerdo al manual

63. 1.2 ¿Realizó una actividad no planificada para esta parte de la sesión?

Marca solo un óvalo.

- Sí
- No

64. 1.3 Realice un comentario respecto a las actividades que no se realizaron, no se realizaron de acuerdo al manual y otras actividades realizadas que no estaban en el manual

65. 1.4.1 Desarrollo planificadas por sesión: Apertura

Marca solo un óvalo.

- No se realiza la actividad
- Se realiza la actividad pero no de acuerdo con el manual
- Se realiza la actividad de acuerdo con el manual
- No aplica

66. 1.4.2 Desarrollo planificadas por sesión: Actividad central 1

Marca solo un óvalo.

- No se realiza la actividad
- Se realiza la actividad pero no de acuerdo con el manual
- Se realiza la actividad de acuerdo con el manual
- No aplica

67. 1.4.3 Desarrollo planificadas por sesión: Actividad central 2

Marca solo un óvalo.

- No se realiza la actividad
- Se realiza la actividad pero no de acuerdo con el manual
- Se realiza la actividad de acuerdo con el manual
- No aplica

68. 1.4.4 Desarrollo planificadas por sesión: Actividad central 3

Marca solo un óvalo.

- No se realiza la actividad
- Se realiza la actividad pero no de acuerdo con el manual
- Se realiza la actividad de acuerdo con el manual
- No aplica

69. 1.5 Haga un comentario sobre la implementación de la apertura

70. 1.6 Haga un comentario sobre la implementación de la actividad central 1

71. 1.7 Haga un comentario sobre la implementación de la actividad central 2

____
____
____
____
____

72. 1.8 Haga un comentario sobre la implementación de la actividad central 3

____
____
____
____
____

73. 1.9 Cierre: Reflexión final

Marca solo un óvalo.

- ◯ No se realiza la actividad
- ◯ Se realiza la actividad pero no de acuerdo con el manual
- ◯ Se realiza la actividad de acuerdo con el manual

74. 1.10 ¿Realizó alguna actividad no planificada en esta parte de la sesión?

Marca solo un óvalo.

- ◯ Sí
- ◯ No

75. 1.11 Realice un comentario sobre las actividades que no se realizaron, las que se realizaron de acuerdo con el manual y otras actividades realizadas que no estaban dentro del manual

____

____

____

____

____

Tiempo de la sesión

76. 1.12.1 Tiempo de inicio

Marca solo un óvalo.

- Usó menos del tiempo asignado
- Cumple con el tiempo asignado
- Excede el tiempo asignado

77. 1.12.2 Tiempo de actividades centrales

Marca solo un óvalo.

- Usó menos del tiempo asignado
- Cumple con el tiempo asignado
- Excede el tiempo asignado

78. 1.12.3 Tiempo de cierre

Marca solo un óvalo.

- Usó menos del tiempo asignado
- Cumple con el tiempo asignado
- Excede el tiempo asignado

79. 1.12.4 Tiempo de la sesión en general

Marca solo un óvalo.

- Usó menos del tiempo asignado
- Cumple con el tiempo asignado
- Excede el tiempo asignado

80. 1.13 Realice un comentario respecto a las actividades que usó menos tiempo del asignado o [illegible] excedió del tiempo asignado

Clima de aula

81. 2.1 En el desarrollo de la sesión, ¿se presentaron situaciones de conflicto?

Marca solo un óvalo.

- Sí
- No *Salta a la pregunta 85*

Sección sin título

82. 2.2 ¿Cuántas situaciones de conflicto se presentaron durante la sesión evaluada?

____

83. 2.3 ¿Todas las situaciones de conflicto fueron resueltas?

Marca solo un óvalo.

- Sí
- No

84. 2.4 En general, para resolver situaciones de conflicto, ¿Cuáles de los siguientes pasos se utilizaron?

Selecciona todos los que correspondan.

- [ ] Entender las necesidades de los estudiantes
- [ ] Empatizar con los estudiantes
- [ ] Expresar la preocupación del Monitor por la conducta del estudiante y sus consecuencias
- [ ] Resolver en forma directiva la situación de conflicto
- [ ] Resolver en forma colaborativa la situación de conflicto
- [ ] Abordar el conflicto, pero dejar en pausa su resolución, para retomarlo después

Sección sin título

85. 2.5 Marque si aborda las siguientes habilidades durante la sesión evaluada

Selecciona todos los que correspondan.

- [ ] Automanejo
- [ ] Conciencia social
- [ ] Opción 3
- [ ] Toma de decisiones
- [ ] Autoconocimiento
- [ ] Habilidades relacionarles

2.6 En relación con mantener la atención y favorecer la participación de los estudiantes, el facilitador...

86. ... preparó el espacio para un adecuado desarrollo de la sesión

Marca solo un óvalo.

	1	2	3	4	5	
Nunca	⬭	⬭	⬭	⬭	⬭	Siempre

87. ... utilizó estrategias para captar y mantener la atención de los estudiantes.

Marca solo un óvalo.

	1	2	3	4	5	
Nunca	⬭	⬭	⬭	⬭	⬭	Siempre

88. 2.7 Pensando en el desempeño durante la sesión evaluada, ¿En cuál de los siguientes estilos docentes se sitúa el facilitador?

Marca solo un óvalo.

- Autoritario
- Autotitativo
- Permisivo
- Negligente

Desempeño

89. 3.1 ¿El/la facilitador/a cuenta con los materiales correspondientes de la sesión?

Marca solo un óvalo.

- No cuenta con todos los materiales necesarios
- Cuenta parcialmente con los materiales necesarios
- Cuenta con todos los materiales necesarios

90. 3.2 El facilitador demuestra manejo de los contenidos de la sesión y por tanto se presenta cc fluidez

Marca solo un óvalo.

- La sesión se realiza con interrupciones de parte del facilitador, y es imposible retomar el diálogo con los estudiantes para completar la sesión. (ej: “Se me olvidó”, “No era así”, “¿Dónde están los materiales?”).
- La sesión se realiza con interrupciones de parte del facilitador, sin embargo, con esfuerzo se pued retomar el diálogo con los estudiantes para completar la sesión
- La sesión se realiza con interrupciones de parte del facilitador, sin embargo, se puede retomar el dialogo fácilmente con los estudiantes para completar la sesión.
- La sesión se realiza sin interrupciones de parte del facilitador, lo cual permite la entrega de los contenidos con fluidez para completar la sesión (Puede apoyarse en el manual, pero sin generar quiebres en el diálogo con los estudiantes)

91. 3.3 Promueve la participación de todos los estudiantes, indique si la facilitadora:

Selecciona todos los que correspondan.

- [ ] Utiliza estrategias equitativas de selección de participantes (ej: bolsa de nombres)
- [ ] Permite la participación de diferentes estudiantes durante la sesión realizando preguntas directivas otorgándole la oportunidad de responder primero a aquellos que no han participado
- [ ] Utiliza estrategias positivas para captar y/o retomar la atención de los estudiantes (ej: dinámicas lúdicas para retomar la atención como cantos, frases y quiebres activos)

4. Relaciones de facilitador

92. 4.1 Mantiene un trato respetuoso y cálido con los estudiantes, indique si la facilitadora realiza alguno de los siguientes comportamientos

Selecciona todos los que correspondan.

- [ ] Realiza escucha activa
- [ ] Utiliza un tono cordial para referirse a los estudiantes
- [ ] Toma las opiniones y las hace parte de la actividad
- [ ] Saluda y se despide de los estudiantes
- [ ] Mantiene una actitud positiva y amable

93. 4.2 Se muestra atento a las necesidades de los estudiantes durante la sesión (Realiza quiebre si observa a los niños/as muy cansados/activos, de ser necesario aborda problemáticas en la sesión o la suspende, etc.)

Marca solo un óvalo.

- ( ) No está atento a esas necesidades
- ( ) Está atento a las necesidades, pero no intenta incorporar a los estudiantes que presentan esas necesidades
- ( ) Está atento y los incorpora promoviendo la participación de todos los estudiantes

94. 4.3 ¿Se encuentra el docente en la sala de clases durante el transcurso de la sesión?

Marca solo un óvalo.

- ( ) Sí
- ( ) No

95. 4.4 Si el docente está en sala, ¿el facilitador promueve la participación del docente durante e
transcurso de la sesión? (ejemplo, le realiza alguna pregunta directa, le solicita ayuda, le ofre
realizar una acción, etc.).

Marca solo un óvalo.

- Sí
- No
