# Supplementary material for: Acceptability, feasibility and fidelity of the culturally adapted version of Unplugged (“Yo Se Lo Que Quiero”), a substance use preventive program among adolescents in Chile: a pilot randomized controlled study
Source: BMC Public Health. 2024 Jul 29;24:2026. doi: 10.1186/s12889-024-19499-2 (PMC11285342; doi:10.1186/s12889-024-19499-2)
Supplement: Supplementary file 4 — Supplementary Material 4 [file 12889_2024_19499_MOESM4_ESM.docx]

**Suppl 4, Table 1: Fidelity of the program according to facilitators and external observers.**

|  | Facilitator | | Observer | |
| --- | --- | --- | --- | --- |
| **Recap of the previous session** | **n** | **%** | **n** | **%** |
| I didn't do the activity | 21 | 7.7 | 15 | 22.7 |
| I performed the activity according to the manual | 235 | 85.8 | 39 | 59.1 |
| I did the activity but NOT according to the manual | 18 | 6.5 | 12 | 18.2 |
| Total general | 274 | 100.0 | 66 | 100.0 |
| **Presentation of the objective** |  |  |  | |
| I didn't do the activity | 6 | 2.2 | 39 | 59.1 |
| I performed the activity according to the manual | 252 | 91.6 | 27 | 40.9 |
| I did the activity but NOT according to the manual | 17 | 6.2 |  |  |
| Total general | 275 | 100.0 | 66 | 100.0 |
| **Aperture** |  |  |  | |
| I didn't do the activity | 1 | 0.4 | 10 | 15.2 |
| I performed the activity according to the manual | 247 | 89.5 | 13 | 19.7 |
| I did the activity but NOT according to the manual | 28 | 10.1 | 43 | 65.2 |
| Total general | 276 | 100.0 | 66 | 100.0 |
| **Core Activity 1** |  |  |  | |
| I didn't do the activity | 3 | 1.2 | 6 | 9.1 |
| I performed the activity according to the manual | 197 | 78.8 | 23 | 34.8 |
| I did the activity but NOT according to the manual | 50 | 20.0 | 37 | 56.1 |
| Total general | 250 | 100.0 | 66 | 100.0 |
| **Core Activity 2** |  |  |  | |
| Not applicable | 3 | 1.3 | 13 | 19.7 |
| I didn't do the activity | 12 | 5.3 | 15 | 22.7 |
| I performed the activity according to the manual | 182 | 79.8 | 15 | 22.7 |
| I did the activity but NOT according to the manual | 31 | 13.6 | 23 | 34.8 |
| Total general | 228 | 100.0 | 66 | 100.0 |
| **Core Activity 3** |  |  |  | |
| Not applicable | 43 | 48.9 | 43 | 67.2 |
| I didn't do the activity | 4 | 4.6 | 9 | 14.1 |
| I performed the activity according to the manual | 32 | 36.4 | 7 | 10.9 |
| I did the activity but NOT according to the manual | 9 | 10.2 | 5 | 7.8 |
| Total general | 88 | 100.0 | 64 | 100.0 |
| **Final Thoughts** |  |  |  | |
| Not applicable | 1 | 0.4 | 0 | 0.0 |
| I didn't do the activity | 13 | 4.8 | 28 | 42.4 |
| I performed the activity according to the manual | 201 | 73.9 | 17 | 25.8 |
| I did the activity but NOT according to the manual | 57 | 21.0 | 21 | 31.8 |
| Total general | 272 | 100.0 | 66 | 100.0 |
| **Closing: Another Unplanned Activity** |  |  |  |  |
| Not applicable | 18 | 29.5 | 0 | 0.0 |
| I didn't do the activity | 5 | 8.2 | 0 | 0.0 |
| I performed the activity according to the manual | 31 | 50.8 | 0 | 0.0 |
| I did the activity but NOT according to the manual | 7 | 11.5 | 0 | 0.0 |
| Total general | 61 | 100 | 0 | 0.0 |

*Note: Total number of sessions=284: Total number of facilitators´ reports=276 (97.2%); Total number of sessions recorded and rated by observers=66 (23.2%)*

**Suppl 4, Table 2: Time sessions**

|  | **Facilitator** | | **Observer** | |
| --- | --- | --- | --- | --- |
| **Start time** | **n** | **%** | **n** | **%** |
| I fulfilled the allotted time | 150 | 54.6 | 14 | 21.2 |
| I exceeded the allotted time | 50 | 18.1 | 34 | 51.5 |
| I used less time than allotted | 75 | 27.3 | 18 | 27.3 |
| Total general | 275 | 100.0 | 66 | 100.0 |
| **Central activity time** | | | | |
| I fulfilled the allotted time | 131 | 48.0 | 22 | 33.3 |
| I exceeded the allotted time | 41 | 15.0 | 4 | 6.1 |
| I used less time than allotted | 101 | 37.0 | 40 | 60.6 |
| Total general | 273 | 100.0 | 66 | 100.0 |
| **Closing time** | | | | |
| I fulfilled the allotted time | 120 | 45.8 | 5 | 7.6 |
| I exceeded the allotted time | 16 | 6.1 | 5 | 7.6 |
| I used less time than allotted | 126 | 48.1 | 56 | 84.8 |
| Total general | 262 | 100.0 | 66 | 100.0 |
| **Overall session time** | | | | |
| I fulfilled the allotted time | 99 | 39.8 | 9 | 13.6 |
| I exceeded the allotted time | 25 | 10.0 | 7 | 10.6 |
| I used less time than allotted | 125 | 50.2 | 50 | 75.8 |
| Total general | 249 | 100.0 | 66 | 100.0 |

*Note: Total number of sessions=284: Total number of facilitators´ reports=276 (97.2%); Total number of sessions recorded and rated by observers=66 (23.2%)*

**Suppl 4, Table 3a: Classroom climate**

|  | **Facilitator** | | **Observer** | | |
| --- | --- | --- | --- | --- | --- |
| **During the Session, were there conflict situations?** | **n** | **%** | | **n** | **%** |
| No | 188 | 68.1 | | 25 | 37.9 |
| Yes | 88 | 31.9 | | 41 | 62.1 |
| Total general | 276 | 100.0 | | 66 | 100.0 |
| **Were all conflict situations resolved?** | | | | | |
| No | 11 | 13.4 | | 14 | 34.2 |
| Yes | 71 | 86.6 | | 27 | 65.8 |
| Total general | 82 | 100.0 | | 41 | 100.0 |
| **I prepared the space for an adequate development of the session** | | | | | |
| Never | 0 | 0.0 | | 22 | 11 |
| Almost never | 0 | 0.0 | | 10 | 5.0 |
| Not Never - Not Always | 21 | 7.6 | | 30 | 15.1 |
| Almost always | 177 | 64.1 | | 32 | 16.1 |
| Always | 78 | 28.3 | | 105 | 52.8 |
| Total general | 276 | 100.0 | | 199 | 100.0 |
| **I used strategies to capture and hold students' attention.** | | | | | |
| Never | 1 | 0.4 | | 10 | 6.7 |
| Almost never | 0 | 0.0 | | 72 | 48.3 |
| Not Never - Not Always | 36 | 13.0 | | 45 | 30.2 |
| Almost always | 192 | 69.6 | | 12 | 8.1 |
| Always | 47 | 17.0 | | 10 | 6.7 |
| Total general | 276 | 100.0 | | 149 | 100.0 |

*Note: Total number of sessions=284: Total number of facilitators´ reports=276 (97.2%); Total number of sessions recorded and rated by observers=66 (23.2%)*

**Suppl 4, Table 3b Students and School teacher participation rated by Observer.**

| **Students' level of attention** | **n** | **%** |
| --- | --- | --- |
| Very low | 0 | 0.0 |
| Low | 11 | 4.0 |
| Medium | 73 | 26.4 |
| High | 154 | 55.8 |
| Very high | 38 | 13.8 |
| Total general | 276 | 100.0 |
| **Level of student participation in activities** | | |
| Very low | 1 | 0.4 |
| Low | 6 | 2.2 |
| Medium | 74 | 27.0 |
| High | 157 | 57.3 |
| Very high | 36 | 13.1 |
| Total general | 274 | 100.0 |
| **Level of teacher involvement** | | |
| Very low | 32 | 11.6 |
| Low | 32 | 11.6 |
| Medium | 70 | 25.4 |
| High | 97 | 35.1 |
| Very high | 45 | 16.3 |
| Total general | 276 | 100.0 |

*Note: Total number of sessions=284: Total number of sessions recorded and rated by observers=66 (23.2%)*

**Suppl 4, Table 4a: Performance rated by the Facilitator.**

| **What level of knowledge about the session did you achieve before implementation?** | **n** | **%** |
| --- | --- | --- |
| I studied it and I knew it perfectly | 240 | 87.0 |
| I studied it and I knew it. but I needed support from the Manual | 36 | 13.0 |
| Total general | 276 | 100.0 |
| **Did you have external interruptions that prevented you from having a smooth session? (e.g. preventive activity against earthquakes. rehearsals of school events. external noises. etc.)** | | |
| No | 224 | 81.2 |
| Yes | 52 | 18.8 |
| Total general | 276 | 100.0 |
| **Did you have the appropriate materials for the session?** | | |
| It has all the necessary materials | 251 | 90.9 |
| It partially has the necessary materials | 25 | 9.1 |
| Total general | 276 | 100.0 |
| **The facilitator demonstrates mastery of the contents of the session and therefore presents himself fluently** | | |
| The session is carried out with interruptions on the part of the facilitator; however, with effort the dialogue with the students can be resumed to complete the session | 6 | 2.2 |
| The session is interrupted by the facilitator; however, the dialogue with the students can be easily resumed to complete the session. | 89 | 32.4 |
| The session is carried out without interruptions on the part of the facilitator, which allows the delivery of the contents with fluidity to complete the session (It can be supported by the manual, but without generating breaks in the dialogue with the students) | 180 | 65.4 |
| Total general | 275 | 100.0 |

**Suppl 4, Table 4b: Performance rated by the Observer.**

| **Does the facilitator have the appropriate session materials?** | **n** | **%** |
| --- | --- | --- |
| It has all the necessary materials | 61 | 92.4 |
| It partially has the necessary materials | 4 | 6.1 |
| It doesn't have all the necessary materials | 1 | 1.5 |
| Total general | 66 | 100.0 |
| **The facilitator demonstrates mastery of the contents of the session and therefore presents himself fluently** | | |
| The session is interrupted by the facilitator; however. the dialogue with the students can be easily resumed to complete the session. | 1 | 1.5 |
| The session is carried out without interruptions on the part of the facilitator. which allows the delivery of the contents with fluidity to complete the session (It can be supported by the manual. but without generating breaks in the dialogue with the students) | 65 | 98.5 |
| Total general | 66 | 100.0 |

**Suppl 4, Table 5: General evaluation by Facilitator.**

| **How would you evaluate the session overall?** | **n** | **%** |
| --- | --- | --- |
| 1 | 1 | 0.4 |
| 2 | 5 | 1.8 |
| 3 | 55 | 20.0 |
| 4 | 177 | 64.4 |
| 5 | 37 | 13.4 |
| Total general | 275 | 100.0 |
| **What aspects would you define as negative about the Session in general?** | | |
| Teacher-student dynamics | 1 | 0.4 |
| Problems in discipline | 7 | 2.5 |
| Time | 35 | 12.6 |
| Students´ participation | 110 | 39.7 |
| Intervention structure | 4 | 1.4 |
| No negative aspect | 8 | 2.9 |
| No comment | 1 | 0.4 |
| No Observation | 111 | 40.1 |
| Total general | 277 | 100.0 |
| **What positive aspects of the Session would you highlight as a whole?** | | |
| Students´attitudes | 144 | 52.0 |
| Favorable Implementation (Methodology) | 5 | 1.8 |
| Participation of school teacher | 22 | 7.9 |
| No Observation | 106 | 38.3 |
| Total general | 277 | 100.0 |

**Suppl 4, Table 6a: Relationship between facilitator, school teacher, and schools reported by Facilitator.**

| **Were there any difficulties in the timetable and access to the school by the school authorities?** | **n** | **%** |
| --- | --- | --- |
| No | 265 | 96.7 |
| Yes | 9 | 3.3 |
| Total general | 274 | 100.0 |
| **Did the teacher or the school put any obstacles in the way of the session?** | | |
| No | 274 | 99.6 |
| Yes | 1 | 0.4 |
| Total general | 275 | 100.0 |
| **Does the teacher of the school show interest in collaborating or participating in the Session?** | | |
| No | 72 | 26.4 |
| Yes | 201 | 73.6 |
| Total general | 273 | 100.0 |

**Suppl 4, Table 6b: Relationship between facilitator, school teacher, and schools reported by observers.**

| **Is attentive to the needs of the students during the session (Breaks if he/she observes the children are very tired/active. if necessary, he/she addresses problems in the session or suspends it. etc.)** | **n** | **%** |
| --- | --- | --- |
| Is attentive to needs, but does not attempt to incorporate students who have those needs | 18 | 27.3 |
| S/he is attentive and incorporates them by promoting the participation of all students | 41 | 62.1 |
| S/he's not attentive to those needs. | 7 | 10.6 |
| Total general | 66 | 100.0 |
| **Is the school teacher in the classroom during the course of the session?** | | |
| No | 10 | 15.2 |
| Yes | 56 | 84.8 |
| Total general | 66 | 100.0 |
| **If the school teacher is in the room, does the facilitator encourage the teacher's participation during the course of the session? (e.g., asks you a direct question. asks for help, offers to take an action. etc.)** | | |
| No | 34 | 51.5 |
| Yes | 32 | 48.5 |
| Total general | 66 | 100.0 |
